# Novel GH115 xylan α‐1,2‐glucuronidases with distinct preferences for xylan‐derived oligomers and polymers

**DOI:** 10.1002/pro.70571

**Published:** 2026-04-08

**Authors:** Savvina Leontakianakou, Simone Balzer Le, Anders Sundin, Andrius Jasilionis, Giang‐Son Nguyen, Anna Nordborg, Lalitha D. Gottumukkala, Carl Grey, Anna Sofia Lewin, Eva Nordberg Karlsson

**Affiliations:** ^1^ Division of Biotechnology and Applied Microbiology, Department of Process and Life Science Engineering Lund University Lund Sweden; ^2^ SINTEF AS, Biotechnology and Nanomedicine Trondheim Norway; ^3^ Centre of Analysis and Synthesis, Department of Chemistry Lund University Lund Sweden; ^4^ Celignis Limited, Mill Court Limerick Ireland

**Keywords:** AlphaFold, dimerization, polymeric xylan, polysaccharide, xylan α‐1,2‐glucuronosidase

## Abstract

Natural polymers are promising sustainable materials for diverse applications. Xylans, major components of hemicellulose, exhibit origin‐dependent substitution patterns that determine their physicochemical properties. Targeted enzymatic modification of these substituents offers a mild and precise approach for tailoring the polymer's characteristics. Glucuronidases (EC 3.2.1.139) and xylan α‐1,2‐glucuronosidases (EC 3.2.1.131) are carbohydrate active enzymes that specifically remove the common glucuronic acid and 4‐*O*‐methyl d‐glucuronic acid side branches of xylans/xylooligosaccharides from different hardwood and softwood sources. These enzymes are, dependent on sequence and structure, occurring in three glycoside hydrolase families (GH4, GH67 and GH115). GH115 predominantly includes xylan α‐1,2‐glucuronosidases, but the number of characterized enzymes is low. In this study, 22 novel GH115 candidates were identified through a combination of automated and manual sequence mining from databases, for evaluation of their production, function and storage possibilities. Twenty enzymes were produced in active form in crude extracts, while only four remained soluble for extended time periods post purification. These four GH115 candidates include first time reported monomeric enzymes, and while all were active on polymeric xylan, their specific activity and activity‐ratio on polysaccharides/oligosaccharides of beechwood xylan differed significantly. Structural models created by AlphaFold3, and different length oligosaccharides were docked in the catalytic clefts. The presence of an additional domain, and the position of a mobile loop affected oligomerization and activity on polymeric substrates, respectively, resulting in differences in specific activity and preference for polymeric substrates.

## 1 INTRODUCTION

Carbohydrate active enzymes (CAZymes) play important roles across many industries due to their versatility and efficiency (Drula et al., 2022). They are used in biofuel production (Mamo et al., 2013), biochemical and material manufacturing (Michaud et al., 2003; Turner et al., 2007), as well as in the production of food, beverages, and pharmaceuticals (Naik & Waghmare, 2020; Trincone, 2020). Because of this broad applicability, they are valuable tools in industrial processing (Bhardwaj et al., 2019; Contesini et al., 2021). Depending on their sequence and function, CAZymes are grouped into different classes, including glycoside hydrolases (GHs), glycosyltransferases (GTs), polysaccharide lyases (PLs), carbohydrate esterases (CEs), and enzymes with auxiliary activities (AAs) (Drula et al., 2022). The largest class in the CAZy database is the GHs, which to date (March 13, 2026) contain 194 families.

Among the various CAZy GH families, only three—GH4, GH67, and GH115—contain reported α‐glucuronidases (EC 3.2.1.139) or xylan α‐1,2‐glucuronosidases (EC 3.2.1.131) (Drula et al., 2022). Xylan α‐1,2‐glucuronosidases catalyze hydrolysis of (1 → 2)‐α‐d‐(4‐O‐methyl) glucuronosyl linkages in the main chain of hardwood xylans, while α‐glucuronidases (EC 3.2.1.139) have a broader definition, hydrolysing α‐d‐glucuronosides to form an alcohol and d‐glucuronate.

From these three families, most GH4 candidates are reported to hydrolyse aryl α‐d‐glucuronides (Mohapatra & Manoj, 2023; Suresh et al., 2003), whereas GH67 glucuronidases act on both short glucuronic acid‐substituted xylo‐oligomers and polymeric glucuronic acid‐containing xylans. Meanwhile, GH115 glucuronidases are more active on polymeric glucuronic acid‐containing xylans (Malgas et al., 2021). Members of GH4 have a NAD^+^‐dependent mechanism, and glucuronidase activity is an exception in this family (2 of 27 characterized enzymes in CAZy) (Mohapatra & Manoj, 2021). GH67 and GH115 members share an inverting catalytic mechanism and are classified in CAZy (visited 08.03.2026) as xylan α‐1,2‐glucuronosidases (EC 3.2.1.131). GH67 enzymes (28 characterized) have recently been reclassified from EC 3.2.1.139 and act only on the non‐reducing end (Drula et al., 2022). GH115 enzymes are predominantly (eight of nine members) endo‐acting xylan α‐1,2‐glucuronosidases that exhibit activity on both the reducing and non‐reducing ends of substrates (Ryabova et al., 2009).

GH115 enzymes are accessory enzymes removing substituents from hardwood and softwood xylan and, less commonly, other biopolymers (e.g., arabinogalactan). In the latter case, a single enzyme, BtGH115A, active on arabinogalactan, is attributed to GH115 (Aalbers et al., 2015). BtGH115A is a monomer in its native form, while the remaining previously characterized GH115 enzymes acting on xylans form homodimers and contain varying numbers of domains, resulting in different molecular weights (Chong et al., 2011; Rogowski et al., 2014; Wang et al., 2016; Wilkens et al., 2022; Yan et al., 2017; Yan et al., 2021). They can act in combination with β‐xylanases, β‐xylosidases, and other accessory enzymes removing branches (arabinofuranosidases, acetyl xylan esterases, etc.) to degrade xylan (Biely et al., 2016; Rhee et al., 2017). In an applied sense, GH115 enzymes can be used together with xylanases in biotechnological processes to saccharify xylan‐containing biomass into fermentable sugars. Sheer debranching of xylans can also be applied to create water‐insoluble forms of xylan (WIS‐xylan). WIS‐xylans form hydrogels and find potential applications in pharmacy, skin care, and food, for example to replace unsustainable petroleum‐derived ingredients. WIS‐xylan could potentially replace silicones in personal care products (especially rinse‐off products), which is important because silicones have raised concerns due to their impact on the aquatic environment and their accumulation in landfills (Horii & Kannan, 2008).

This study aims to expand the available enzyme toolbox by selecting new putative GH115 candidates for cloning and production, via manual sequence similarity screening from the CAZy database (nine candidates), as well as via high‐throughput screening from public and proprietary sources (eight candidates), to investigate structure–function interactions of successfully produced candidates and gain further insight into factors important for their enzymatic catalysis.

## 2 MATERIALS AND METHODS

### 2.1 Biodiversity sequence datasets for sequence mining

#### 2.1.1 Grass enrichment culture

A proprietary sequence dataset for sequence mining was generated from digestates originating from anaerobic digesters processing agriculture waste samples. The digestate samples were further enriched by continuous digestion under anaerobic, mesophilic conditions (37°C) for 3 months with a daily feeding rate of 2.5 g/L volatile solids loading in a 5 L continuous stirred tank reactor system. The feedstock used was late cut grass with an arabinoglucuronoxylan content of 18% (product information from Celignis Ltd., Limerick, Ireland). A sample from this anaerobic enrichment was used for DNA isolation followed by sequence analysis and library construction. Total DNA was extracted following the extraction protocol for sediments described in Kotlar et al. (Kotlar et al., 2011). Evaluation of DNA after isolation was done using Qubit and Nanodrop for quantification and by agarose gel (0.8%) stained with GelRed® (Biotium) for molecular weight determination. Shotgun sequencing libraries were made following the Nextera XT DNA Library Preparation Kit (Illumina cat. FC131‐1024) and sequenced by the Illumina MiSeq instrument. The generated sequence dataset was stored locally on a high‐performance computer and organized to aid sequence mining.

#### 2.1.2 Actinobacteria strain collection

A proprietary sequence dataset of draft genome sequences of isolates was generated, with the techniques above, from a marine Actinobacteria strain collection originating from the Trondheim fjord. This collection is known to include novel strains and be a source of various novel enzymes (Cordas et al., 2022; Králová et al., 2021; Sandoval‐Powers et al., 2021; Stepnov et al., 2021).

#### 2.1.3 Publicly available sequence databases

The databases used for gene selection include NCBIGenBank (https://www.ncbi.nlm.nih.gov/), CAZy (https://www.cazy.org), MarDB, and MarFun. MarDB and MarFun are public databases available through the Marine Metagenomics portal (MMP) of University of Tromsø (UiT) (https://sfb.mmp2.sigma2.no/). MarDB includes all marine microbial genomes regardless of level of completeness, and MarFun is a manually curated marine fungi genome database. Both databases can be downloaded from https://sfb.mmp2.sigma2.no/download-2/ and used for sequence mining. The versions used in the data mining that generated the described results are MarDB 6^1^ and MarFun3^2^.

### 2.2 GH115 gene mining

Gene mining was performed implementing a combination of manually curated and automated mining workflow‐based approaches.

#### 2.2.1 Gene selection via the CAZy database

Sequences indexed in the CAZy database (https://www.cazy.org) as attributed to GH115 were selected. Reference sequence sets were obtained from the CAZy “Structure” and “Characterized” sequence index sections and were downloaded from the UniProtKB database Swiss‐Prot section, and GenBank for initial sequence set assembly.

Initial sequence sets were accumulated from publicly deposited GenBank sequences (https://www.ncbi.nlm.nih.gov/) indexed in the CAZy database, following sequence attribution criteria to the requested CAZyme systematic group (Drula et al., 2022). Candidate sequences were first analyzed for the presence of signal peptides using SignalP v.5.0 (Almagro Armenteros et al., 2019). Signal peptides were omitted before further sequence analysis. The HHpred server (Zimmermann et al., 2018), applying pairwise comparison of HMMs against PDB_mmCIF70_12_Oct, Pfam‐A_v35 and NCBI_Conserved_Domains(CD)_v3.18 databases, was used to determine sequence domain organization. Comparison with reference sequences was performed by aligning with ClustalW in MEGA X (Kumar et al., 2018). If sequences contained domains other than the catalytic/family domain, they were subjected to parallel comparison and alignment of only the catalytic domain sequences. The initial selection of potential enzyme candidates was based on a multiple sequence alignment (MSA), evaluating conserved motif pattern(s) and overall catalytic domain identity with reference sequences. Catalytic domain sequence identity no <20% at a sequence coverage of 40–50% was considered as a minimum criterion for enzyme candidate selection. This was combined with the presence of conserved catalytic amino acids in the predicted catalytic domain of enzyme candidates. Additional criteria were domain organization (N‐terminal catalytic domain (pfam15979) followed by two domains was considered as canonical for GH115), origin from a genome sequenced microorganism (candidate sequences from organisms with fully annotated genome sequences were favored). Econiches occupied by source organisms expected to contain relevant genes were also considered.

Solubility of each selected enzyme candidate in *Escherichia coli* was predicted using the Protein‐Sol tool (available at http://protein-sol.manchester.ac.uk) under the default model parameters. This tool is predicting protein solubility based on the observation of a bimodal distribution of protein solubilities for *E. coli* proteins in cell‐free expression (using 35 sequence‐based properties), for which the average soluble protein is 45% on a scale from 0 to 100%, where higher solubility corresponds to a higher value. The prediction is based on integration of protein sequence physicochemical properties and a model trained on a dataset of experimentally characterized protein solubility statistics (Hebditch et al., 2017).

#### 2.2.2 Data mining from alternative databases

Automated datamining was made from the databases MarDB, MarFun as well as from in house resources at SINTEF. The data mining workflow was based on profile Hidden Markov Models (pHMMs) of the GH115 (Profile HMM PF15979, Pfam), the HMMER software suit (version 3.4), and the eggNOG‐mapper (version 2.1.7) software. The datasets used for mining are described above. A sequence Similarity Network (SSN) approach was applied using local BLAST (version 2.16.0+) with visualization in Cytoscape (version 3.9) to shortlist the most relevant candidates for experimental characterization. For constructing of the clustered tree shown in Figure 2, a multiple sequence alignment (MSA) was generated using ClustalO (version 1.2.4). The MSA was used as input for FastTree (version 2.1.11) and PHYLIP (version 3.697 using its internal tools SEQBOOT, PROTDIST, NEIGHBOR, CONSENSE) to generate a consensus tree from the 100 bootstrapped trees. The tree was edited and annotated with the sequence alignment using TreeViewer version 2.2.0. Protein structures of candidate enzymes were predicted using local ColabFold (version 1.5.2). Predicted structures were compared and aligned with those in the Protein Data Bank (PDB) using Foldseek (version 9‐427df8a). Structural information was used to confirm the candidates obtained from the sequence‐based selection when predicted structures resembled known ones. Finally, protein solubility was predicted using the local Protein‐Sol tool, described above (https://protein-sol.manchester.ac.uk/software) under the default model parameters (Hebditch et al., 2017).

### 2.3 Preparation of genetic constructs for expression

Genes encoding putative GH115 proteins, from the manual selection were de novo synthesized by Gene Universal (USA) not altering the native codon landscape and only, if necessary, adjusting sequences in the context of selected restriction endonuclease cloning sequences. An affinity tag encoding sequence was incorporated at the N‐terminus of the respective GH115 target gene, containing His_6_‐tag and TEV protease recognition sites (Kumar et al., 2018). Cloning was done (*Nde*I‐*Xho*I sites used) in the pET‐21b(+) vector (Merck). Expression constructs were validated by sequencing.

GH115 candidate genes obtained through the automated workflow‐based data mining selection, along with two positive control GH115 encoding genes [encoding *wts*Agu115A from an uncultured bacterium (PDB ID: 7PUG) (Wilkens et al., 2022) and *Bo*Agu115A from *Bacteroides ovatus* (PDB ID: 4C90) (Rogowski et al., 2014)] were synthesized by GenScript (The Netherlands). This included codon optimization, cloning into the expression vector pET21b (+), and fusion of a His_6_‐tag encoding sequence at the 3′ end.

Validated expression constructs were transformed into *Escherichia coli* BL21(DE3) competent cells (Merck) through heat‐shock transformation.

### 2.4 Primary evaluation of GH115 candidates

#### 2.4.1 Enzyme production

*Escherichia coli* clones expressing the genes coding for GH115 enzymes were cultivated in triplicates in 96‐well polypropylene plates with U‐shaped bottom (96PPU) (Greiner Bio‐One). Cultures were grown overnight in Lysogeny‐Broth (LB) medium at 900 rpm, 85% relative humidity (RH) and 37°C. Precultures were transferred to a new 96PPU plate containing 80 μL reduced Hi‐YE medium per well, supplemented with ampicillin (200 μg/mL) per well using a 96 pin replicator. The medium contained: Na_2_HPO_4_·2H_2_O, 12.3 g/L; KH_2_PO_4_, 4.29 g/L; NH_4_Cl, 0.43 g/L; NaCl, 0.71 g/L; glucose, 2.86 g/L; yeast extract, 2.86 g/L; MgSO_4_, 1.86 mM; Fe(III)‐citrate, 99 μM; H_3_BO_3_, 21 μM; MnCl_2_, 46 μM; EDTA, 9.7 μM; CuCl_2_, 3.8 μM; Na_2_MoO_4_, 4.4 μM; CoCl_2_, 4.5 μM; and zink acetate, 15 μM. The plate was incubated for 20–22 h at 900 rpm, 85% RH and 37°C.

Gene expression was induced using 40 μL induction medium (glycerol (99.5%), 30.2 g/L; yeast extract, 24 g/L; and IPTG, 1.5 mM) supplemented with ampicillin (200 μg/mL) per well. Cultures were incubated for 24 h at 900 rpm and 16°C. Cells were harvested by centrifugation at 2619×*g* for 25 min and 4°C.

#### 2.4.2 Cell lysis

The pellets were lysed by addition of 120 μL deionized water supplemented with 0.05 mg/mL lysozyme and 2.5 U/mL Benzonase® Nuclease (Millipore) per well.

At this stage, another positive control was included. 3 μg/mL of recombinant α‐methyl‐glucuronidase 115A from *Bacteroides ovatus* (NATE‐1452, Creative Enzymes) was added per control well containing *E. coli* BL21(DE3) pUC19 cell pellet. After one freeze–thaw cycle (at −20°C), lysates were incubated for 1–1.5 h at 37°C and 500 rpm to complete enzymatic lysis.

#### 2.4.3 Activity screening

Following lysis, 50 μL of crude cell lysate was transferred to a flat‐bottom 96‐well polystyrene plate. GH115 activity was determined using beechwood xylan (P‐XYLNBE, Megazyme) at a final substrate concentration of 4 mg/mL. For this assay, 100 μL of beechwood xylan solution (6 mg/mL in 50 mM sodium phosphate buffer, pH 6.5) was added to each lysate. Reactions were incubated at 40°C for 22–24 h. Two readouts were applied. The first approach was to screen for an increase in turbidity. The release of 4‐*O*‐methyl d‐glucuronic acid reduces xylan solubility, leading to precipitation. Absorbance at 560 nm was measured at 0, 1, 2.5, 5, and 25 h.

Secondly, the actual release of 4‐*O*‐methyl d‐glucuronic acid was quantified using liquid chromatography–mass spectrometry (LC–MS/MS). For this, 100 μL of reaction mixture was transferred to a polypropylene 96 deep‐well plate. Reactions were quenched by adding four parts of ethanol (400 μL) per part of reaction mixture, followed by freezing at −20°C. Samples were centrifuged at 4816×*g*, for 20 min at 4°C to remove debris, then centrifuged again at 21,475×*g* for 15 min to remove precipitated proteins. Supernatants were further diluted in 80% methanol prior to analysis. The LC–MS/MS itself was performed using an Agilent 6490A triple quadrupole mass spectrometer coupled to an Agilent 1290 HPLC system (Agilent, Santa Clara, CA, USA). Chromatographic separation employed a Waters Aquity BEH amide column (2.1 × 150 mm, 1.7 μm; Waters, Milford, MA, USA) with gradient elution from 90% to 10% acetonitrile in 50 mM ammonium acetate over 6 min at flow rate of 0.3 mL/min. The mass spectrometer was operated in negative ionization mode and multiple reaction monitoring (MRM) using three transitions for the target analyte.

### 2.5 Protein production and purification of short‐listed candidates

A 1% pre‐inoculum of each *E. coli* recombinant culture, pre‐cultivated overnight in LB medium at 37°C with 100 μg/mL ampicillin as a selection marker, was introduced into 100 mL of LB medium. The cultures were incubated with shaking at 37°C until reaching an optical density (OD) at 600 nm of 0.6–0.8. Then, they were induced with 1 mM IPTG and further incubated for 14–16 h at 16°C or 26°C.

Cells were then harvested, lysed by sonication, and centrifuged at 26,000×*g*, at 4°C to separate soluble proteins from cell debris. The resulting soluble fractions were purified by nickel affinity chromatography using an ÄKTA start protein purification system (GE Healthcare Bio‐Sciences, Sweden) equipped with a HisTrap™ High‐Performance column (1 mL, Cytiva). The system was equilibrated, and unbound proteins were washed out with a binding buffer 50 mM HEPES‐NaOH pH 7.4/RT, containing 500 mM NaCl, 50 mM imidazole, and 5% (v/v) glycerol. The His‐tagged protein bound to the resin were eluted performing single‐step elution with a buffer composed of 50 mM HEPES‐NaOH, pH 7.4/RT, containing 500 mM NaCl, and 500 mM imidazole. Protein concentration was determined spectrophotometrically by measuring absorbance at 280 nm, while purity was estimated using sodium dodecyl sulfate polyacrylamide gel electrophoresis (SDS‐PAGE).

### 2.6 Nano differential scanning fluorimetry (nanoDSF) for thermal unfolding (T_*m*_) at different pH values

The Prometheus NT 48 nanoDSF (NanoTemper Technologies, Germany) instrument was used to determine the protein unfolding. In detail, 0.2 g/L enzyme were prepared in pH 4.4, 5.6, 6.4, 7.0, 7.8, 8 (McIlvaine buffer system). Each sample (10 μL) was loaded to standard capillaries of the equipment. The intrinsic fluorescence was then monitored by measuring the emission at wavelengths of 330 nm and 350 nm while subjecting the samples to a temperature gradient ranging from 20°C to 90°C, with a temperature increase of 1°C per minute. For samples with unclear unfolding temperature (e.g., Agu115A_10), the temperature gradient was extended to 4°C per minute to facilitate the transition. All data analysis was performed using the PR.Therm.Control software (Version 2.0, Germany), and the *T*_*m*_ was extracted from the first derivative of the 350/330 absorbance ratio.

### 2.7 Production of GXOs with GH30

Glucuronoxylooligosaccharides (GXOs) were produced from beechwood xylan (Megazyme). A pretreatment was performed using Glucuronoxylanase 30A from *Bacteroides ovatus* (*Bo*Xyn30A) (NZYtech, Portugal) (Salas‐Veizaga et al., 2021). In brief, 4% (w/v) of beechwood xylan was dissolved in a 50 mM sodium phosphate buffer (pH 7.0). Based on Rogowski et al. (2015) (Rogowski et al., 2015), 1 mg/mL BSA was added to the reaction mixture along with *Bo*Xyn30A (0.01 g/L), and the mixture was incubated under shaking at 37°C for 12 h. The reaction was terminated via heating at 100°C for 5 min.

### 2.8 Enzymatic reaction products

The reaction products from the enzyme activity were analyzed with HPAEC‐PAD, ICS 6000, Dionex (Thermo Scientific), as described in Leontakianakou et al. (Leontakianakou et al., 2024). A CarboPac PA200 analytical column (250 mm × 3 mm, 5.5 μm) equipped with a respective guard column (50 × 3 mm) was used for the analysis of samples before and after the enzymatic treatment. The mobile phase consisted of 100 mM NaOH and a gradient of sodium acetate of 0–100 mM during the first 10 min after which it was kept constant at 100 mM until the end of the run at 23 min for the beechwood and GXOs analysis. 4‐*O*‐methyl‐d‐glucuronic acid of 0.6–4.8 mM was used as standard for identification and quantification. Excel was used for all functions, equations, fittings, and graphs.

### 2.9 Factorial design (DoE) for activity optimization on beechwood xylan

Design of experiments (DoE) was performed using MODDE13 (Sartorius Stedim Data Analytics). Temperature and pH were investigated as experimental factors using a screening design, followed by an optimization design to map the response surface and identify conditions yielding improved enzymatic activity within the tested design space. Experiments at different temperatures 20, 36, 45, 53, and 70°C and pH 3, 5, 6, 7, and 9 were conducted. The buffering system used was McIlvaine for the range between pH 3–7 and CHES for pH 9. Reactions were run for 1 h, with 0.5% w/v beechwood xylan (Megazyme) and 0.1 g/L of enzyme and terminated by heating at 100°C for 10 min. The production of 4‐*O*‐methyl‐d‐glucuronic acid was determined with the HPAEC‐PAD method as described above and a heat map was generated indicating the best conditions and the effect of these parameters on the enzymes.

### 2.10 Specific activity determination on beechwood xylan and fractionated beechwood

Enzymatic reactions were performed under conditions determined from the T_m_ analysis and DoE results. Due to increased viscosity at higher substrate concentrations, all reactions were conducted using 0.1% (w/v) beechwood xylan as the substrate in McIlvaine buffer at pH 7.5, 6.0, 4.4, and 6.4 for BbAgu115A, FAgu115A, Agu115A_1b, and Agu115A_10, respectively. Reactions were carried out at 38°C. Reactions were initiated by the addition of enzyme. Samples were collected at 0, 0.5, 1, 2, 3, 5, 7, 10, 30, and 1440 min. For Agu115A_10, samples were collected at 0, 5, 10, 30, 60, 120, 180, and 1440 min to ensure accurate determination of the initial reaction rate. Reactions were terminated by heating the samples at 100°C for 5 min. All reactions were performed in duplicate, and products were analyzed using HPAEC‐PAD as described above. 4‐O‐methyl‐d‐glucuronic acid was used as an external standard for quantification.

### 2.11 Size exclusion chromatography (SEC) and OMNISEC analysis

Analytical gel filtration was employed for the determination of the molecular weight and the native form of *Bb*Agu115A, *F*Agu115A, Agu115A_1b and Agu115A_10. All samples were run on Superdex 200 Increase 10/300 GL (Cytiva) with degassed McIlvaine buffer of their optimal pH based on their T_*m*_ at room temperature. The column was re‐equilibrated with the pH optimum buffer for each enzyme between every run. Protein concentrations varied between runs and were typically in the micromolar range. Gel filtration calibration was performed using the Cytiva Gel Filtration Calibration Kit HMW (code 28403842) according to manufacturer's instructions Standard markers (ferritin, aldolase, conalbumin and albumin) were used for the column the calibration and blue dextran for the calculation of void volume The apparent partition coefficient (K_av_) was calculated for all peaks.

The oligomeric state of the samples in solution was further evaluated using an OMNISEC RESOLVE/REVEAL system (Malvern Panalytical, UK). Prior to injection, samples were filtered through 0.2 μm centrifugal filters (Millipore) and analyzed in triplicate. The system consisted of the OMNISEC RESOLVE module equipped with a Superdex 200 Increase 10/300 GL column (Cytiva), integrating a pump, degasser, autosampler, and column oven, coupled to the OMNISEC REVEAL multi‐detector module. The detectors included right‐angle light scattering (RALS, 90°), low‐angle light scattering (LALS, 7°), differential refractive index, a viscometer, and a diode‐array UV/VIS spectrometer. Detector normalization was performed with bovine serum albumin (Thermo Fisher Scientific, USA).

Analyses were carried out at a flow rate of 0.5 mL/min using a mobile phase of 20 mM Tris–HCl, 150 mM NaCl, adjusted to pH 8.0 and degassed prior to use. Protein concentrations varied between 0.5 and 1.2 mg/mL. Data acquisition and processing were performed using OMNISEC v11.41 software (Malvern Panalytical). Reproducibility of the results was confirmed by overlaying the chromatographic profiles from triplicate injections.

### 2.12 Structural analysis

Protein structures were predicted using AlphaFold 3 (https://alphafoldserver.com/). For all enzymes, the model 0 was selected for further calculations. Molecular modifications, molecular mechanics minimizations, and molecular dynamics simulations were performed with Schrödinger release 2025–2, using OPLS4 force field (Schrödinger, USA). The xylopentaose ligand was obtained from the X‐ray crystal structure of GH115 α‐1,2‐glucuronidase *wts*Agu115A from an uncultured bacterium (PDB ID: 7PUG) in complex with xylopentaose (Wilkens et al., 2022). Only the third xylose residue could accommodate branching with a glucuronic acid (GlcA) side chain, as it provided protein contacts while avoiding steric hindrance. This placed the GlcA substituent in a cavity with significant protein contact. Therefore, the glucuronic acid branch was added manually to the third xylose and was energy optimized in the catalytic cavity. Molecular graphics were generated using PyMOL Graphics System (Schrodinger, 2015).

## 3 RESULTS

### 3.1 GH115 candidate selection

#### 3.1.1 Sequence selection from the CAZy database

Two main approaches were implemented to prioritize and characterize new candidates from GH115 for the enzymatic removal of the 4‐*O*‐methyl‐d‐glucuronic acid substituent from complex beechwood xylan. The first included manually curated sequence mining of publicly deposited gene‐sequences (https://www.ncbi.nlm.nih.gov/) indexed under GH115 in the CAZy database. The selection of candidates from this database was based on a comparison of 1132 sequences with six characterized enzyme reference sequences (RefSeq: WP_015011011.1; GenBank: EDO10816.1; RefSeq: WP_008767247.1; RefSeq: WP_006858276.1; GenBank: ABD81015.1; RefSeq: WP_005320908.1).

**FIGURE 1:**
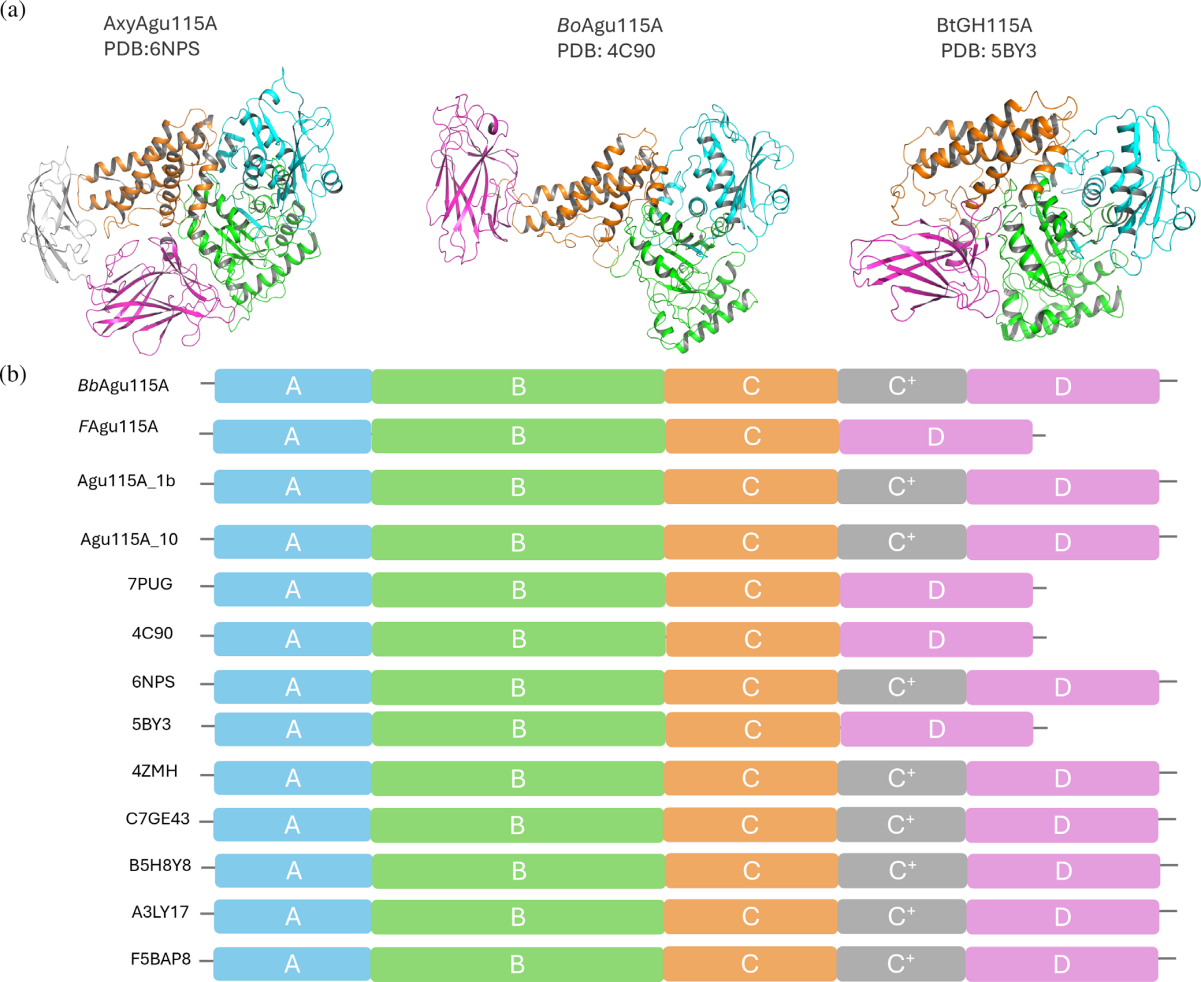
(a) Domain‐colored model for three crystal structures with different architecture. (b) Domain chart for all the characterized proteins in the family (indicated by their PDB or Uniprot codes), including the ones described in this work (indicated by their protein names). Catalytic PF15797 domain specific for GH115 translates to residues 164–496 for BbAgu115A, which corresponds to domains A and B. These domains are conserved in all GH115 structures. Whereas PF17829, residues 752–920 relates to domain D with one β‐sheet on C^+^ domain for BbAgu115A (Figure S1).

The selection of genes was then picked by combining phylogeny with conserved features of the deduced amino acid sequence of the respective catalytic domain and resulted in a shortlist of 37 sequences that demonstrated no <20% identity of the deduced amino acid sequence to the reference sequence catalytic domains at 40–50% sequence coverage. Based on the additional selection criteria, 28 of those sequences were omitted, leaving a final list comprising nine putative GH115 encoding sequences (Table 1). The omission was based on incomplete genome sequencing of the microbial source and/or domain organization predicted at low confidence. Selected putative GH115 enzymes were typically encoded by Gram‐negative soil mesophiles. Signal peptide sequence presence was predicted for all nine candidates, showing that the selected enzymes were secreted from the cytoplasm. Alignment of their respective sequences with reference sequences confirmed that the candidates included the GH115 domain (pfam 15,979) and C‐terminal domain (pfam 17,829) commonly appended to GH115 enzymes. Additional domains, N‐terminal of the pfam 17,829 domain, were also distinguished in the selected sequences as shown in Figure 1.

**TABLE 1:** Enzyme candidates from manual selection in the CAZy and NCBI databases.

Enzyme designation	Species	Strain	Thermotolerance	Genome RefSeq:	Calculated Mw (kDa)	Protein sol prediction (%)
*Bb*Agu115A	*Belliella baltica*	BA134	Mesophile	NC_018010.1	105.2	53.4
*Wf*Agu115A	*Wenyingzhuangia fucanilytica*	CZ1127	Mesophile	NZ_CP014224.1	82.87	38.0
*Pt*Agu115A	*Paenibacillus terrae*	HPL‐003	Mesophile	NC_016641.1	80.09	37.9
*Ps*Agu115A	*Paraflavitalea soli*	5GH32‐13	Mesophile	NZ_CP032157.1	93.16	36.9
*Ot*Agu115A_1	*Opitutus terrae*	PB90‐1	Mesophile	NC_010571.1	104.8	34.1
*Ot*Agu115A_2	*Opitutus terrae*	PB90‐1	Mesophile	NC_010571.1	93.88	26.1
*Mm*Agu115A_1	*Mucilaginibacter mali*	G2‐14	Mesophile	NZ_CP054139.1	109.4	33.4
*Mm*Agu115A_2	*Mucilaginibacter mali*	G2‐14	Mesophile	NZ_CP054139.1	107.5	38.6
*F*Agu115A	*Flavobacteriaceae* bacterium	3519–10	Psychrophile^a^	NC_013062.1	93.28	44.1

^a^ Not cultivated, but isolated from a psychrophilic environment.

#### 3.1.2 Automated sequence selection from alternative databases

In a second approach, selection of new GH115 enzymes was performed using a data mining pipeline to look for new candidates in a number of alternative databases. The dataset from the metagenomic library originating from the grass enrichment culture contained around 17,000 ORFs which were annotated with putative functions. Further data input included the sequence dataset from the in‐house Actinobacteria strain collection (at SINTEF), in addition to the publicly available MarFun and MarDB databases.

After manual curation to filter the relevant GH115 candidates (having α‐glucuronidase activity) using a profile HMM obtained from the Pfam database as input, a list of 1360 HMM hits was generated. These were further shortlisted to 13 candidates for experimental characterization (Table 2): one from the grass enrichment culture in two variants (with and without signal peptide), seven from MarDB, one from MarFun, and three from the Actinobacteria strain collection.

**TABLE 2:** Enzyme candidates from data mining workflow‐based selection.

Designation	Source ID	Best hit (NCBI/GenBank)	Pairwise identity (%)	Query coverage (%)	Description of hits	Organism^a^	MW (kDa)^b^	Protein‐sol prediction (%)
Agu115A_1a	CELIGNIS_contig_157_3_1_a_ ^c^	MDY0239689	100	100	MAG: glycosyl hydrolase 115 protein, partial [Bacteroidales bacterium]	b	109	39.6
Agu 115A_1b	CELIGNIS_contig_157_3_1_b_ (without predicted signal peptide)^c^	MDY0239689	100	100	MAG: glycosyl hydrolase 115 protein, partial [Bacteroidales bacterium]	b	107	39.6
Agu 115A_2	mardb_Glyco_hydro_115_CAAHFG010000001.1_MMP5207384_5^d^	WP_136079369	100	96	glycosyl hydrolase 115 [Pontiella desulfatans]	b	105	32.5
Agu 115A_3	mardb_Glyco_hydro_115_NZ_PHQQ01000004.1_MMP08099983_5^d^	WP_103474844	100	100	MULTISPECIES: glycosyl hydrolase 115 protein [Arenibacter]	b	78	47.8
Agu 115A_4	mardb_Glyco_hydro_115_NZ_RJUK01000001.1_MMP10363429_1^d^	WP_170162829	100	100	glycosyl hydrolase 115 protein [Marinimicrobium koreense]	b	140	53.1
Agu 115A_5	mardb_Glyco_hydro_115_QKDK01000103.1_1^d^	WP_092476472	55	98	glycosyl hydrolase 115 protein [[Clostridium] polysaccharolyticum]	b	111	41.8
Agu 115A_6	mardb_Glyco_hydro_115_QKDK01000167.1_4^d^	WP_099467189	54	95	glycosyl hydrolase 115 protein [Konateibacter massiliensis]	b	84	47.8
Agu 115A_7	mardb_Glyco_hydro_115_RHGE01000048.1_MMP13439265_3^d^	TAJ11798	100	98	hypothetical protein DMA11_15510 [Marinilabiliaceae bacterium JC017]	b	107	32.9
Agu 115A_8	mardb_Glyco_hydro_115_VOQF01000012.1_MMP12500376_4^d^	WP_146950033	100	96	glycosyl hydrolase 115 protein [Metabacillus litoralis]	b	87	52.1
Agu 115A_9	marfunV3_Glyco_hydro_115_DF238783.1_MMP00002584_5^e^	XP_012187910	100	97	hypothetical protein PHSY_001894 [Pseudozyma hubeiensis SY62]	f	121	35.9
Agu 115A_10	P01‐F09_Glyco_hydro_115_scaffold106_size17956_ (−3)^f^	WP_030627125	99	100	MULTISPECIES: glycosyl hydrolase 115 protein [Streptomyces]	b	115	42.1
Agu 115A_11	P06‐G04_Glyco_hydro_115_scaffold15_size78841_ (−2)^f^	WP_306081212	93	100	MULTISPECIES: glycosyl hydrolase 115 protein [Streptomyces]	b	114	38.9
Agu 115A_12	P26‐D05_Glyco_hydro_115_scaffold135_size24402_len24402_cov60.5670_ (−3)^f^	WP_006379935	91	99	MULTISPECIES: glycosyl hydrolase 115 protein [Streptomyces]	b	106	45.1

^a^ b: bacterial; f: fungal.

^b^ Calculated MW including His6 tag (Gasteiger et al., 2005).

^c^ Grass enrichment culture.

^d^ Publicly available database MarDB.

^e^ Publicly available database MarFun.

^f^ Internal SINTEF sequence database from an Actinobacteria strain collection.

**FIGURE 2:**
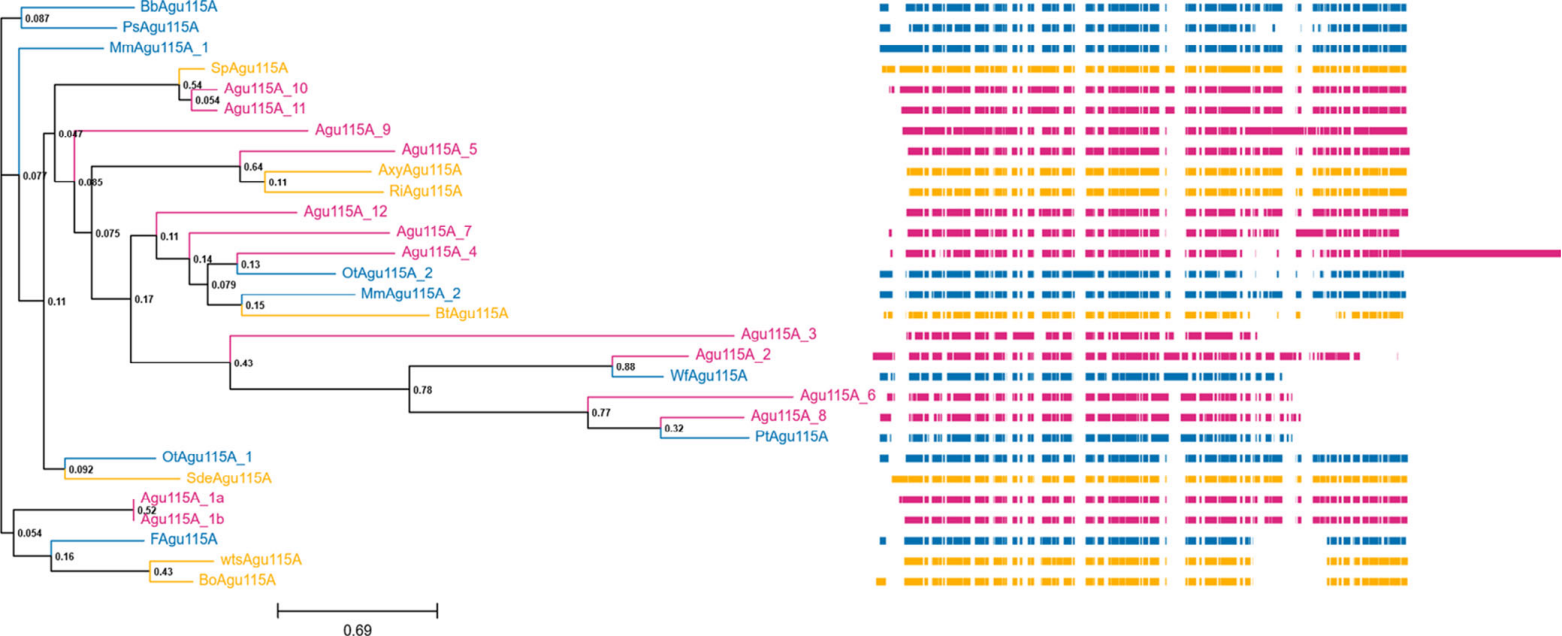
Clustering of sequences obtained from manual selection (9 sequences) and data mining workflow (13 sequences) based on multiple sequence alignment (MSA, blue: Sequences from manual selection, red: Sequences from data mining workflow, yellow: Sequences of reference GH115 structures). The bootstrap values are shown at each of the nodes. The right panel shows the MSA where the color blocks indicate conserved regions of the sequences.

The calculated molecular weight (Mw) of the selected GH115 enzyme sequences varied between 78 and 140 kDa, reflecting differences in domain composition (Figure S2‐6). The estimated solubility in *E*. *coli* was expected to be average soluble based on the Protein‐Sol tool estimation. Based on a multiple sequence alignment of the 22 candidates and the two reference GH115, a dendrogram was generated (Figure 2) to show the phylogenetic differences between the selected amino acid sequences.

Sequence‐wise the candidates from both approaches clustered with each other in different distinctive clades while sharing many conserved regions with the two references *Bo*Agu115A (PDB ID: 4C90) (Rogowski et al., 2014) and *wts*Agu115A (PDB ID: 7PUG) (Wilkens et al., 2022).

### 3.2 Initial activity screening of GH115 candidates

**FIGURE 3:**
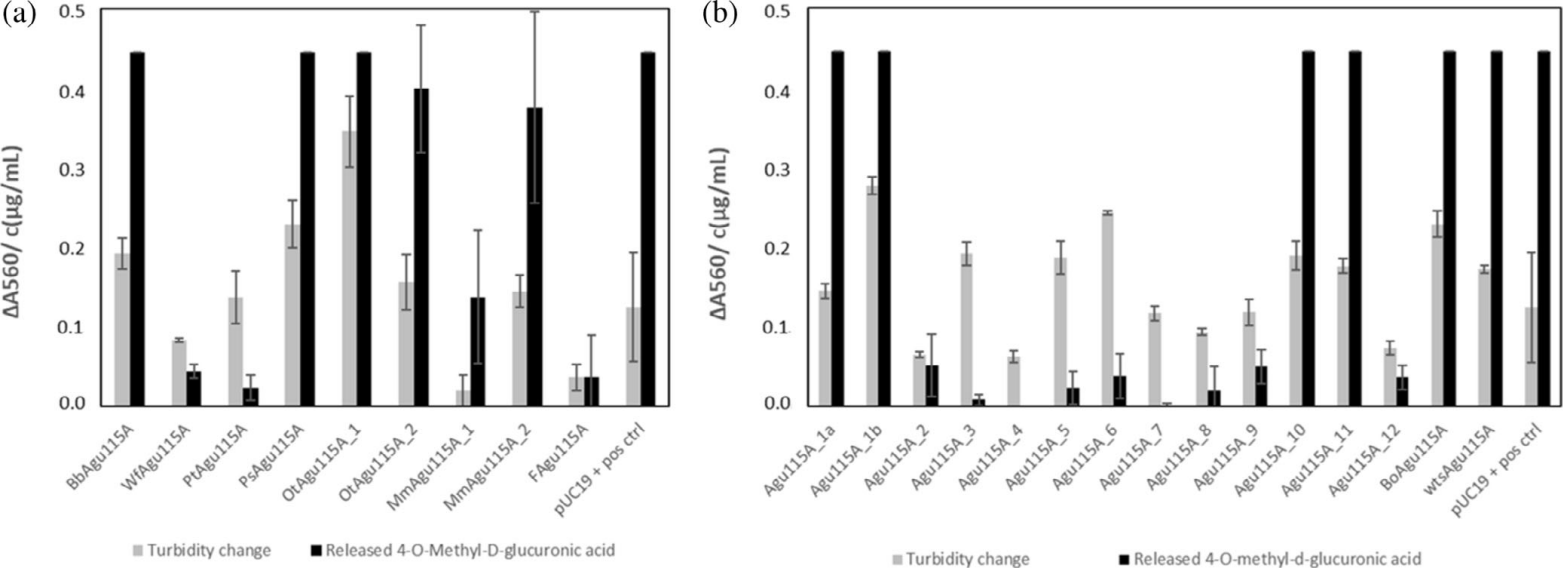
Initial evaluation of GH115 candidates. GH115 activity in crude cell lysates was evaluated by an increase in turbidity upon incubation with beechwood xylan (expressed as the difference in A_560_ after 25 h incubation compared to the time 0 control; gray bars) and resulting released 4‐O‐methyl‐d‐glucuronic acid in μg/mL (black bars). Note: 0.45 μg/mL was the upper detection limit. (a) Produced GH115 candidates from CAZy selection. Each enzyme is represented by the designation given in Table 1. (b) Produced GH115 candidates and controls from the data mining pipeline. The designation of each candidate enzyme is shown. The reference enzymes are represented by constructs producing the two enzymes BoAgu115A and wtsAgu115A. Values for the negative control (lysate of E. coli strain transformed with pUC19) have been subtracted in all cases. The positive control was recombinant α‐methyl‐glucuronidase 115A from Bacteroides ovatus (NATE‐1452, Creative Enzymes) dissolved in the pUC19 lysate. Data represent averages and standard deviations from three replicas. Values on the y‐axis apply to both detection methods. As a reference, the negative control strain (E. coli BL21(DE3) pUC19) released 0.055 μg/mL 4‐O‐MeGlcA and the background turbidity at 560 nm was 0.12.

Expression of all candidate genes in *E. coli* BL21 (DE3) led to production of GH115 enzymes, which after cell lysis resulted in an increase in turbidity upon incubation with beechwood xylan. A release of the product (4‐*O*‐methyl‐d‐glucuronic acid) exceeding background from the pUC19 negative control was detected for all but two candidates. The highest concentrations of the product compound were detected in nine crude lysates containing *Bb*Agu115A, *Ps*Agu115A, *Ot*Agu115A_1, OtAgu115A_2, MmAgu115A_2, Agu115A_1a, Agu115A_1b, Agu115A_10, and Agu115A_11, in addition to the lysates of the positive controls (Figure 3). Soluble protein production was also confirmed by SDS‐PAGE (data not shown). Differences in predicted solubility of the chosen candidates were, however, difficult to relate to the activity results of the screening (Figure 3) within the solubility range selected (26.1–53.4%).

### 3.3 Activity, pH optima and thermostability of the GH115 candidates

Based on the selection criterium of showing activity on beechwood xylan in crude extracts, the active candidates (Figure 3) were purified by metal ion affinity chromatography to allow further characterization. This, however, showed that several of the pure target enzyme candidates were aggregation‐prone (visible as precipitate and loss of activity), including the highly active PsAgu115A, OtAgu115A_1 and Agu115A_11 from the extracts (Figure 3). Five candidates remained soluble, of which four were selected for further characterization: BbAgu115A, FAgu115A, Agu115A_1b, and Agu115A_10. (Agu115A_1a was omitted as the only difference to the selected Agu115A_1b was the presence of a signal peptide).

Production of the four candidates, stable in purified form in solution, was further evaluated under two different IPTG induction concentrations (0.05 and 1 mM) and two post‐induction incubation temperatures, 18°C and 26°C, showing that while a decreased inducer concentration was not benefiting overall production, a decrease in temperature resulted in improved production of the two candidates BbAgu115A and Agu115A_10 (data not shown).

**FIGURE 4:**
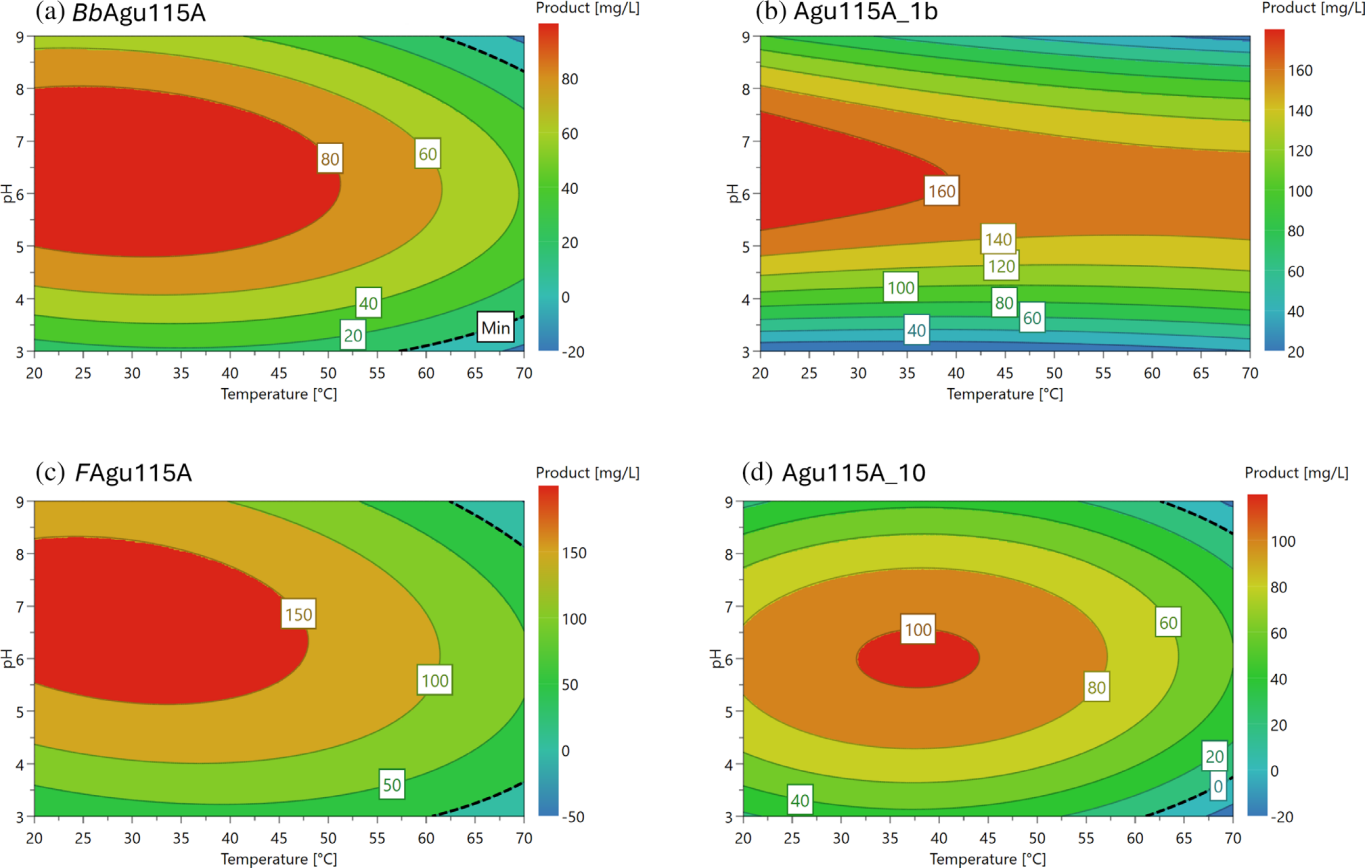
Heat maps of the enzyme targets illustrating the optimal reaction conditions, for the release of *4‐O‐methyl‐d‐glucuronic acid* from polymeric beechwood, in terms of temperature and pH. The X‐axis corresponds to pH, and Y‐axis temperature, the color gradient, refers to the predicted amount of released product. The warmer colors (orange/red) represent higher product release, and cooler colors (blue/green) indicate lower product release.

A classic DoE scheme was used to find the purified enzymes' temperature range and pH optima for activity on beechwood xylan, where the product (4‐*O*‐methyl‐d‐glucuronic acid) concentration was used as response in 2D heatmaps (Figure 4). *Bb*Agu115A and *F*Agu115A exhibited the most similar profiles, with activity across the temperature range (20–50°C) and (20–47°C), respectively, with product release in a relatively broad pH window between pH 5–8 and pH 5.5–8.5, respectively. Notably, *F*Agu115A achieved higher level of product release than *Bb*Agu115A, reaching up to 150 mg/L of 4‐*O*‐methyl‐d‐glucuronic acid. Agu115A_1b appeared to be more influenced by pH than by temperature, showing some activity in the complete temperature range tested within the pH range 5.5–7.5. Finally, Agu115A_10 demonstrated the most restricted optimal conditions, with activity limited to 30–45°C, at pH 6.

The melting temperature (*T*_*m*_) of each protein was subsequently determined at different pHs (Table 3). *Bb*Agu115A, *F*Agu115A, Agu115A_1b, and Agu115A_10 were most stable at pH 7.0, 6.4 (5.6–4.4), and 4.4, respectively, with corresponding *T*_*m*_ values of 44.15 ± 0.05°C, 49.3 ± 0.3°C, 46.35 ± 0.45°C, and 49.2 ± 0.3°C. This shows enzyme‐specific differences in pH‐optima for thermostability, with two of the enzymes, *Bb*Agu115A and *F*Agu115A, being more stable at a higher pH.

**TABLE 3:** Nanoscale differential scanning fluorimetry (nanoDSF) measurements for the monitoring of *T*_*m*_ of the four shortlisted enzymes at different pH values.

pH	*Bb*Agu115A	Agu115A_1b	*F*Agu115A	Agu115A_10
3.2	N.D.	41.35 ± 0.05	N.D.	42.0 ± 0.7
4.4	N.D.	46.35 ± 0.05	N.D.	49.2 ± 0.3
5.6	34.55 ± 0.95	46.35 ± 0.45	N.D.	48.4 ± 0.7
6.4	43.35 ± 0.05	45.75 ± 0.45	49.3 ± 0.3	46.9 ± 0.5
7	44.7 ± 0.0	45.45 ± 0.15	46.85 ± 0.05	45.2 ± 0.6
7.8	44.15 ± 0.05	45.05 ± 0.05	43.8 ± 0	39.7 ± 0.4
8	43.95 ± 0.05	44.7 ± 0.0	43.55 ± 0.05	38.6 ± 0.4
w. ligand	57.2 ± 0.2	46.5 ± 0.8	47.85 ± 0.05	46.7 ± 0.9

Abbreviations: N.D., not determined; w. ligand, beechwood xylan 1% (w/v) at the pH showing the highest *T*_*m*_.

Influence of the substrate on thermostability was confirmed for the enzyme *Bb*Agu115A as the addition of beechwood xylan at the pH where the enzyme was most thermostable, led to an increase of T_*m*_ of approximately 13°C, indicating enzyme–substrate interactions that stabilized the protein. Such significant stabilization was, however, not observed for the other candidates in the presence of the ligand. In contrast, FAgu115A exhibited a slight decrease in Δ*T*_*m*_ in the presence of the ligand, suggesting a possible stabilization of the unfolded state of the protein (Waldron & Murphy, 2003). It can thus be emphasized that the influence of substrate on the thermostability is enzyme dependent.

### 3.4 Oligomeric state

The four enzyme candidates were analyzed with size exclusion chromatography (SEC) at the pH showing highest thermostability (Table 4). This analysis was expanded by running OMNISEC from Malvern Panalytical at pH 8 for measurement of absolute molecular weight, molecular size, and intrinsic viscosity (Table 4). The resulting values were 113, 220, and 109 kDa for the *Bb*Agu115A, *F*Agu115A, and Agu115A_1b, respectively, whereas no defined results were obtained for Agu115A_10 (due to loss in the filtration step prior to analysis).

**TABLE 4:** Physicochemical parameters of the four proteins obtained from OMNISEC RESOLVE/REVEAL analysis.

	*Bb*Agu115A			*F*Agu115A				Agu115A_1b			Agu115A_10	
OMNISEC	Peak 1	Peak 2	Peak 3	Peak 1	Peak 2	Peak 3	Peak 4	Peak 1	Peak 2	Peak 3	Peak 1	Peak 2
RV (ml)	13.64	14.71	16.39	13.43	15.44	16.28	18.35	13.48	14.65	16.4	13.6	18.5
Mw (g/mol)	490,605	273,028	113,073	561,103	220,515	192,451	66,690	449,713	236,860	109,695	499,568	72,526
Mp (g/mol)	473,877	262,164	110,730	513,970	208,040	188,032	60,823	454,994	229,708	108,318	470,385	49,235
Mw/Mn	1.002	1.003	1.002	1.01	1.005	1.002	1.007	1	1.003	1.001	1.007	1.038
IVw (dL/g)	0.1926	0.5784	0.0342	0.1672	0.0762	0.161	0.1401	0.7771	0.1908	0.0317	1.7594	0.2747
Rhw (nm)	11.24	10.99	3.91	9.17	5.24	7.32	5.2	16.83	8.66	3.76	23.84	5.11
Frac. Of sample (%)	5.5	3.7	90.8	21.6	39.9	12.6	25.9	2.4	3.2	94.4	46.5	53.5
Measured conc. (mg/mL)	0.57	0.57	0.57	0.15	0.15	0.15	0.15	1.04	1.04	1.04	0.0091	0.0091
Recovery (%)	48	48	48	30.8	30.8	30.8	30.8	100	100	100	1.8	1.8
Theoretically calculated Mw (kDa) from amino acid sequence with tag	107.45			95.55				107.26			115.36	

*Note*: RV: retention volume measured at peak maximum. Mw: weight average molecular weight from the molecular weight distribution; Mp: molecular weight at the peak apex; Calculated Mw: calculated using http://web.expasy.org/protparam/ based on the provided amino acid sequence of the expression construct; Mw/Mn: dispersity; IVw: weight average intrinsic viscosity; Rhw: weight average hydrodynamic radius; Frac. of sample: fraction of the sample in this peak expressed as a percent of the total area of all the analyzed peaks; Measured conc.: measured concentration of the peak; Recovery: recovery of sample based on RI peak area and input dn/dc compared to the input sample concentration (definitions from the OMNISEC system User Guide MAN0550‐06‐EN, provided on demand).

The monomeric state of *Bb*Agu115A was consistent between SEC and OMNISEC analysis. The protein eluted as two weak, high molecular weight species followed by a major peak, corresponding predominantly to the monomeric form (~90%), with minor amounts of dimer (~4%) and a possible tetramer (~5%). The dispersity values indicated that the sample was essentially monodisperse and homogeneous. Analysis of Agu115A_1b showed a very similar pattern. OMNISEC revealed 94.4% monomer, 3% dimer, and 2% tetramer, whereas SEC performed at the optimal pH detected only monomeric and dimeric species.

For *F*Agu115A, partial sample loss in the filtration step prior to OMNISEC analysis resulted in approximately 30% recovery and a complex elution profile with four peaks. The main peak accounting for 39.9% of the recovered mass corresponded to a dimer and was homogeneous and monodisperse. Additional peaks corresponded to monomer, possible tetramer, and degradation products. However, SEC analysis at pH 6 yielded a dominant peak corresponding to a dimer, suggesting that pH influences the oligomeric state of this enzyme in solution.

Finally, Agu115A_10 exhibited very low recovery in OMNISEC (<2%), with peaks consistent with tetrameric and degraded species, which may indicate sensitivity to host cell proteases (Rozkov & Enfors, 2004). SEC traces were also weak, indicating an ongoing aggregation process including monomeric and degraded forms, preventing a clear determination of the enzyme's biological oligomeric state.

### 3.5 Activity on fractionated and polymeric beechwood Xylan

**FIGURE 5:**
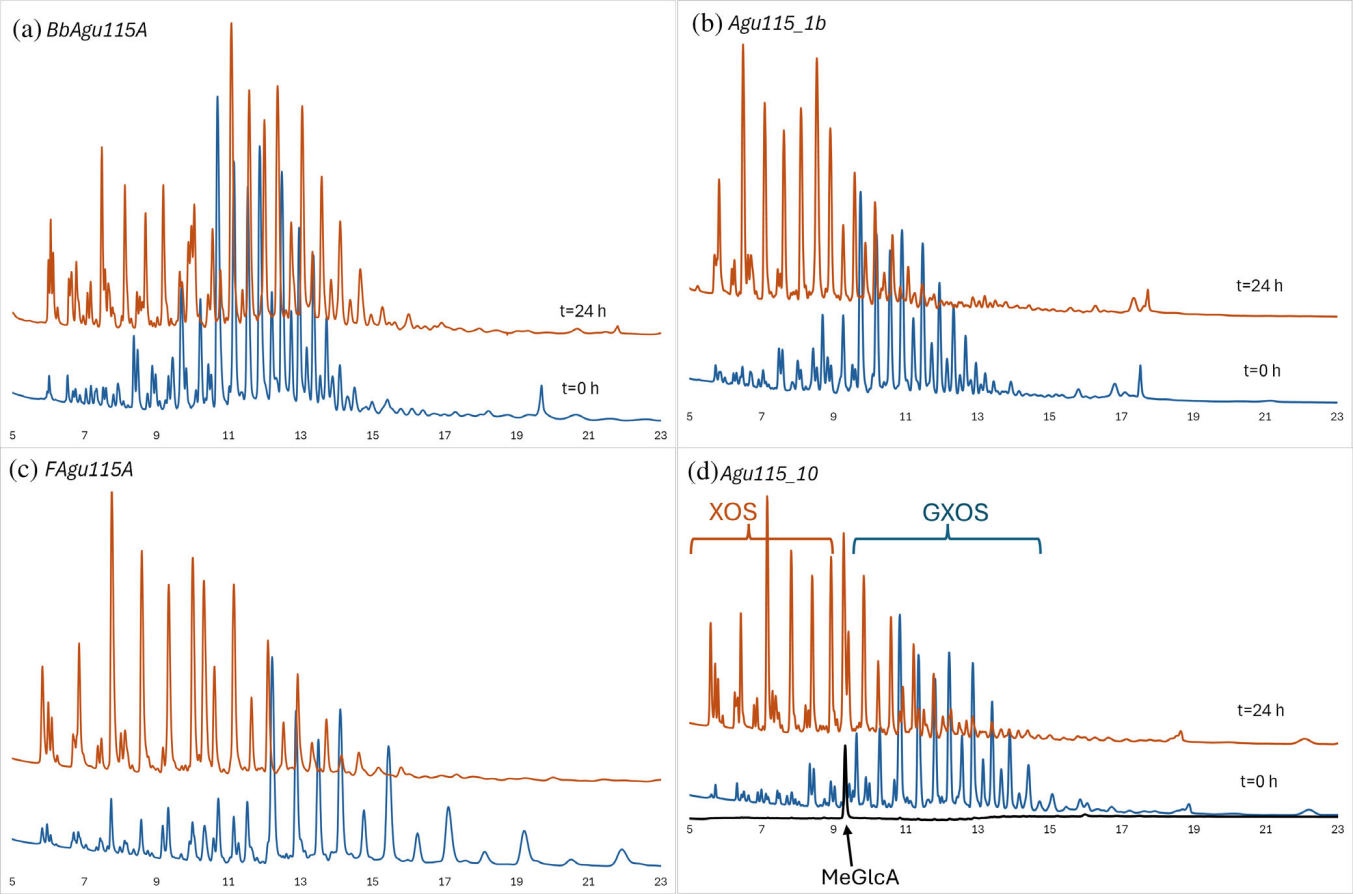
HPAEC‐PAD Chromatograms of glucuronoxylooligosaccharide reaction products. Panels show reactions of (a) *Bb*Agu115A, (b) Agu115A_1b, (c) *F*Agu115A, and (d) Agu115A_10 with fractionated beechwood glucuronoxylooligosaccharides. Chromatograms are shown at 0 h (blue) and 24 h (orange). MeGlcA was used as standard (black).

The enzymes' activity on oligosaccharides was investigated and compared to the activity on the polymeric substrate. Polymeric beechwood xylan was fractionated after treatment with *Bo*Xyn30A glucuronoxylanase. Xylooligosaccharides of varying lengths carrying 4‐O‐methyl‐d‐glucuronic acid (MeGlcA) branches were generated through enzymatic pretreatment with the GH30‐8 glucuronoxylanase CtXyn30A. This enzyme specifically recognizes MeGlcA substitutions on the xylan backbone and cleaves the glycosidic bond two residues toward the reducing end from the substituted xylose, where the MeGlcA is positioned at the −2 subsite relative to the scissile bond (Puchart et al., 2021). The oligosaccharide fraction was then used as a substrate to evaluate debranching enzyme activity. The three enzymes, Agu115A_1b, *F*Agu115A, and Agu115A_10, showed strong activity on glucuronoxylooligosaccharides (Figure 5b–d). After 24 h of reaction, the peak spectrum changed, showing that glucuronoxylooligosaccharide peaks were replaced by peaks corresponding to unsubstituted xylooligosaccharides. In contrast, the activity of *Bb*Agu115A was low, and glucuronoxylooligosaccharides were still present after 24 h (Figure 5a), indicating a stronger preference for the polymeric substrate. This is supported by the chromatographic profile, where it appears that peaks at retention times exceeding 15 min are reduced whereas earlier eluted peaks remain intact, suggesting that the enzyme is only active at GXOS of higher DP.

This pattern was clearly visible after calculation of the specific activity (Table 5). Among the shortlisted candidates, both *F*Agu115A and *Bb*Agu115A had a preference for polymeric over oligomeric beechwood xylan, but the dimeric *F*Agu115A exhibited 15 x higher specific activity on the polymer than the monomeric *Bb*Agu115A. The monomeric Agu115A_1b had similar activity levels for either polymeric or oligomeric substrate. For the oligosaccharide substrate, the activity of Agu115A_1b was in the same range as observed for *F*Agu115A and Agu115A_10. Agu115A_10 was, however, the only enzyme that had a preference for the oligosaccharide, and in this case, the activity on the polymer was 7.7 times lower (Table 5).

**TABLE 5:** Specific activity of the four proteins on 0.1% (w/v) beechwood xylan and 0.1% (w/v) fractionated beechwood xylan.

	Specific activity on beechwood xylan (μM/min)/mg	Specific activity on oligosaccharides from beechwood xylan, degraded by GH30 (μM/min)/mg
*Bb*Agu115A	1638 ± 52	711 ± 105
*F*Agu115A	25,973 ± 3924	4944 ± 678
Agu115A_1b	5622 ± 1129	6250 ± 1581
Agu115A_10	519 ± 16	4000 ± 16

### 3.6 Conserved residues and structure analysis

**FIGURE 6:**
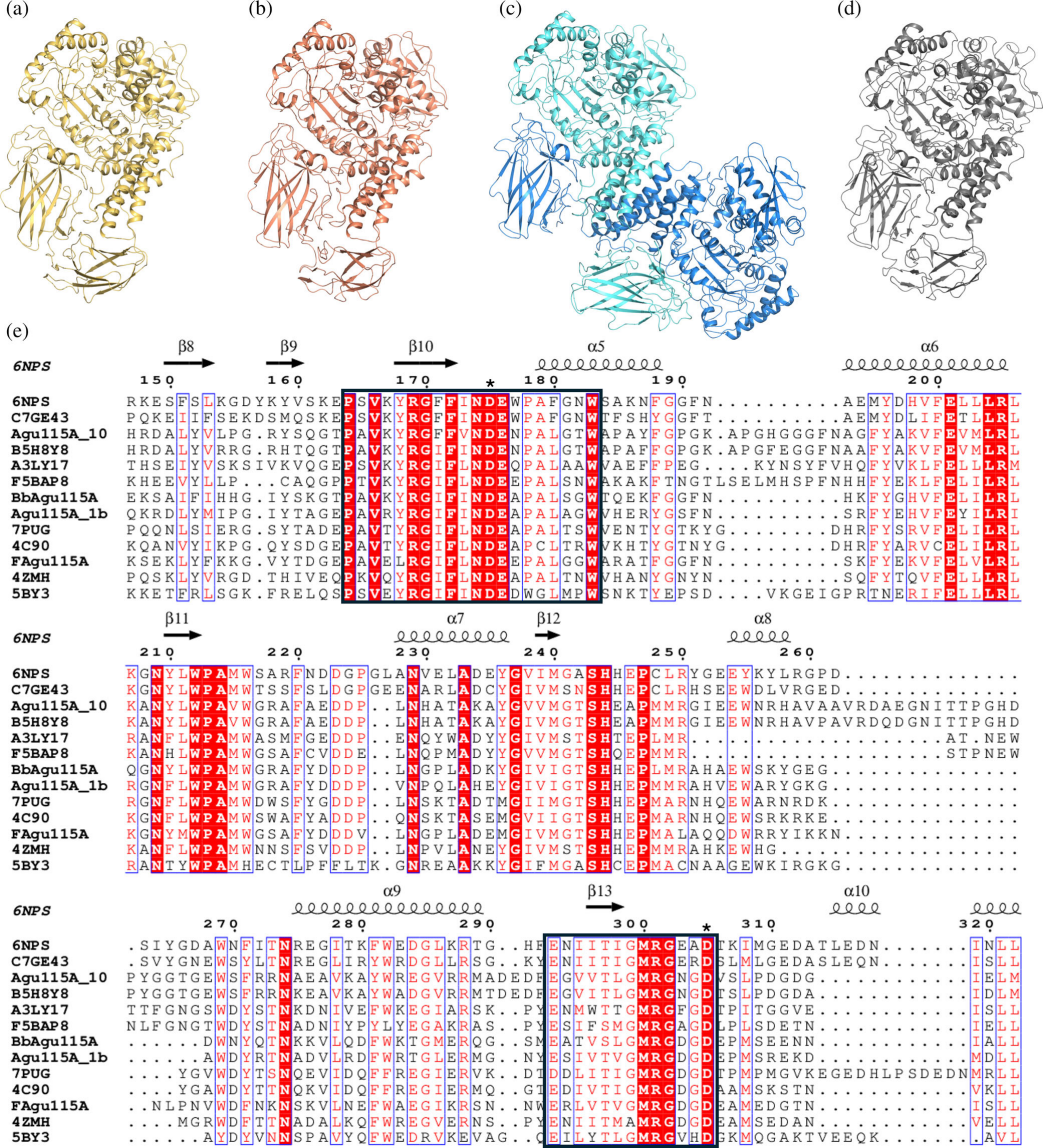
Model of individual proteins. (A) BbAgu115A (yellow), (b) Agu115A_1b (orange), (c) FAgu115A (blue chain A, cyan chain B) and (d) Agu115A_10 (gray). (e) Multiple sequence alignment of all characterized or structurally determined GH115 proteins listed in CAZy, including the candidates described in this study. The secondary structure of the five‐domain enzyme AxyAgu115A (30) (PDB ID: 6NPS) is shown above the alignment as a structural reference. Conserved regions containing residues previously reported in the literature as putative catalytic residues are indicated by black frames, and the specific residues are marked with an asterisk.

Modeling of the enzymes by AlphaFold3 (Figure 6) showed that *Bb*Agu115A, Agu115A_1b, and Agu115a_10 were composed of five domains each, whereas *F*Agu115A has four (in accordance with the domain chart in Figure 1b).

To get further insights in the main similarities and differences between structurally investigated GH115‐members, all structure‐determined GH115 candidates (five crystal structures, four deposited models, and four proteins from this work) were aligned using ClustalW. MSA analysis revealed amino acids conserved across all GH115 sequences (Figure 6e), that based on current literature data include amino acids crucial for catalysis (Kolenová et al., 2010; Rogowski et al., 2014; Ryabova et al., 2009; Wilkens et al., 2022), along with their surrounding residues. The monomeric units of the GH115 proteins were superimposed, showing a preserved fold within the catalytic domains (CDs), with the root mean square deviation (RMSD) of the helical domains being between 0.2 and 1.6 Å. All structures exhibited a common fold with well‐preserved secondary structure near the ligand binding site.

### 3.7 Molecular dynamics (MD) and ligand recognition

**FIGURE 7:**
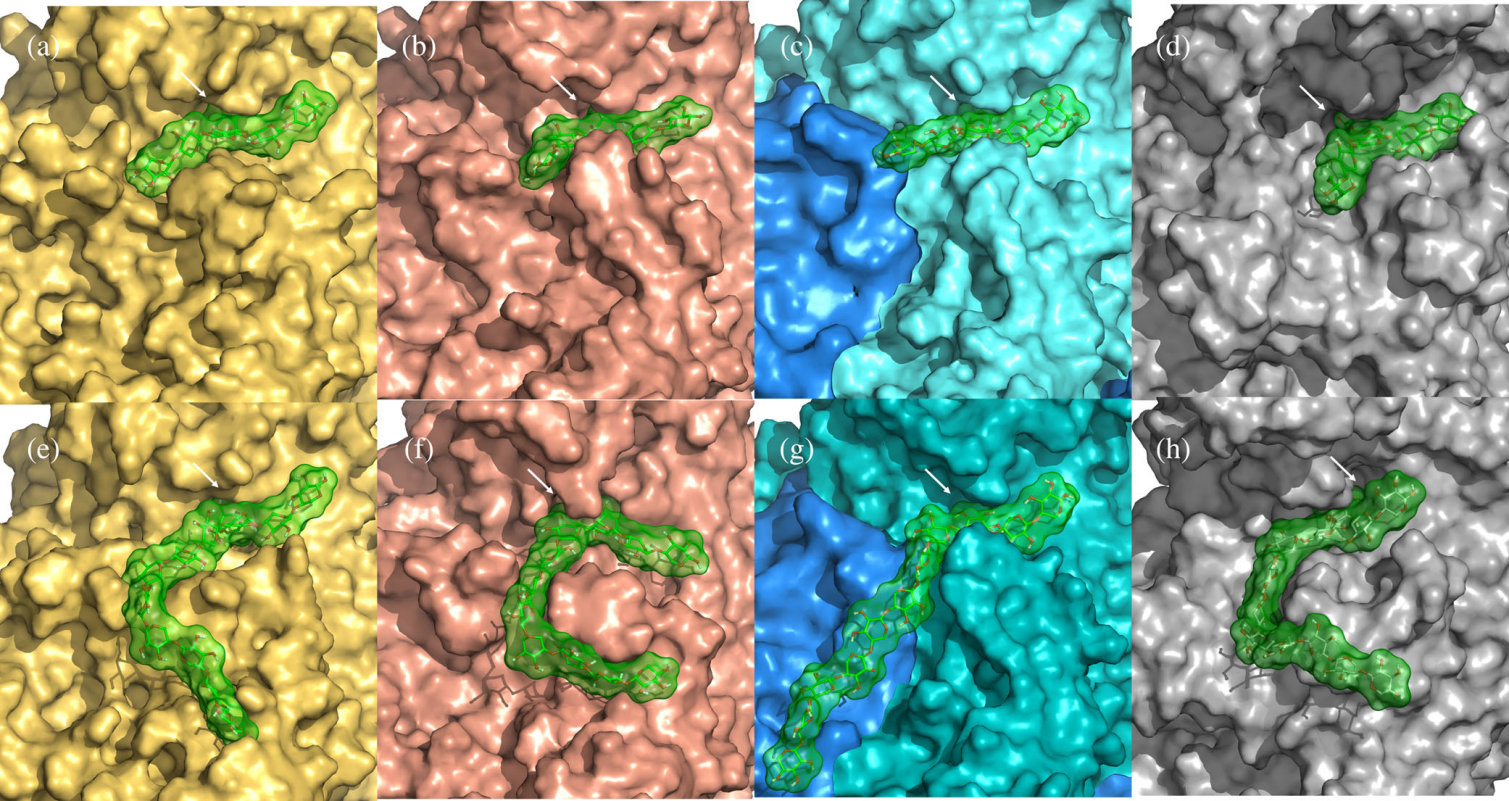
Surface representation of protein–ligand complexes after 100 ns MD simulation. Panels (a–d) depict an oligomeric chain containing a X_5_ ligand with GlcA linked to the third xylose moiety. Panels E–H show an X_11_ chain with GlcA on the third xylose. GlcA shown by a white arrow in all panels. From left to right: *Bb*Agu115A is shown in yellow, Agu115A_1b in salmon, and *F*Agu115A is represented with each protomer colored blue and cyan and Agu115A115_10 in gray.

Molecular dynamics (MD) simulations of 100 ns were performed to evaluate substrate recognition for all the proteins using both an oligosaccharide substrate with five xylose units (Figure 7a–d) and a longer substrate with 11 xylose units (Figure 7e–h). Across all eight simulations, the ligands remained stably bound, and the GlcA moiety consistently adopted a twist‐boat conformation within the binding pocket, corresponding to an estimated ring strain of about 4 kcal/mol. Aromatic residues contributed to the recognition of the oligomeric xylopentaose ligand, stabilizing interactions at both ends of the substrate. In *Bb*Agu115A, a mobile loop containing a tryptophan residue (Trp620) rapidly lost interaction with its corresponding xylose, correlating with the enzyme's low specific activity on fractionated beechwood xylan (Figure 7a). This observation suggests that minimum three xylose units required for efficient catalysis.

In simulations with the longer xylose chain, *F*Agu115A, *Bb*Agu115A, and Agu115A_1b all exhibited similar behavior: past residue X₇, the chain initially engaged with the solvent before settling into an additional binding site. In *Bb*Agu115A, Agu115A_1b, and Agu115A_10 (Figure 7e,f,h), the ligand twisted around a loop (from Gln621‐Ile631, numbering corresponds to *Bb*Agu115A) in distinct patterns for each enzyme, indicating available space for accommodating longer xylose chains in *Bb*Agu115A. In contrast, *F*Agu115A exhibited a more linear recognition mode, where the GlcA moiety was positioned in the binding pocket of one protomer while xylose moieties X₆–X₁₁ were recognized by the second protomer, suggesting that dimerization can facilitate binding of extended substrates (Figure 7f).

## 4 DISCUSSION

Efficient screening of multiple target enzymes for substituent removal from polymeric xylan can be challenging, as this is often related to reduced solubility of the substrate. For this purpose, an assay for turbidity measurements was developed (described in materials and methods) and combined with direct LC–MS/MS detection of released 4‐*O*‐methyl‐d‐glucuronic acid. This double screen was used as an alternative to the previously used coupled spectrophotometric measurement of uronic acid release after oxidation to glucarate with concomitant reduction of NAD^+^ to NADH (Rogowski et al., 2014; Wang et al., 2016; Wilkens et al., 2022). Several targets were identified from the combined screening that could potentially function as competent xylan α‐1,2‐glucuronosidases, but more than half of the candidates were not stable in solution after purification. These enzymes included *Ps*Agu115A, *Ot*Agu115A (1 and 2), *Mm*Agu115A (1 and 2), and Agu115A_11 (Figure 3). It should, however, be noted that these candidates could still be considered for industrial applications where protein purification is not required, as the released 4‐*O*‐methyl‐d‐glucuronic acid in the screening showed that these enzymes were active in the crude extract. Shortlisted candidates, selected for further characterization, were both active in the screening and stable in solution post‐purification. These included the four enzymes *Bb*Agu115A, *F*Agu115A, Agu115A_1b, and Agu115A_10. Of these, *F*Agu115A showed relatively low activity in the double screen (low turbidity increase coupled with a low increase in 4‐*O*‐methyl‐d‐glucuronic acid). Nevertheless, the sequence similarity to *Bo*Agu115A (Rogowski et al., 2014) and wtsAgu115A (Wilkens et al., 2022), that served as positive controls in the screening, and the protein concentration and stability after purification led to selection of *F*Agu115A for additional testing, showing that the enzyme had high specific activity, and that the clone in the screening must have been low in production of recombinant enzyme. The Protein‐Sol tool was used to originally select genes with a higher likelihood to be produced in soluble form in *E. coli* (Hebditch et al., 2017) but did not distinguish successfully purified candidates from those that precipitated after purification.

To further investigate the structure–function relationships, structural models were made of the four selected GH115 enzymes and specific activities were determined. The specific activity was determined by direct measurement of the released 4‐*O*‐methyl‐d‐glucuronic acid from the natural substrate beechwood xylan by HPAEC‐PAD (Leontakianakou et al., 2024). Determination of the kinetic parameters for the release of methylated glucuronic acid was challenging, as it is a major substituent of beechwood xylan (Hromádková et al., 2005), and its release caused viscosity effects. The increase in viscosity, after releasing higher product concentrations, made sampling inefficient and introduced significant deviations among replicates, limiting the methodology to use of lower concentrations of beechwood xylan (<1% (w/v)), to allow reproducible quantification of the released 4‐*O*‐methyl‐d‐glucuronic acid, shown as specific activity. *F*Agu115A exhibited the highest specific activity of the four enzymes, suggesting that this four‐domain dimer configuration performed best on polymeric xylan, but the enzyme also displayed significant activity on mixed oligomers of varying degrees of polymerization. *Bb*Agu115A and Agu115A_1b share a similar five‐domain monomeric structural architecture; however, Agu115A_1b displayed approximately 3.4‐fold and 8.8‐fold higher specific activity on beechwood xylan and fractionated beechwood xylan, respectively. Agu115A_1b also displayed specific activity in the same range for the polysaccharide and oligosaccharide mix, while *Bb*Agu115A preferred the polysaccharide. Agu115A_10 was the only enzyme with preference for oligomeric substrate. As the method for activity determination is new, direct activity comparison is limited to the enzymes in the current study, but structural comparisons and MD simulations (not previously performed for GH115 candidates) revealed interesting differences between the four enzymes.

**FIGURE 8:**
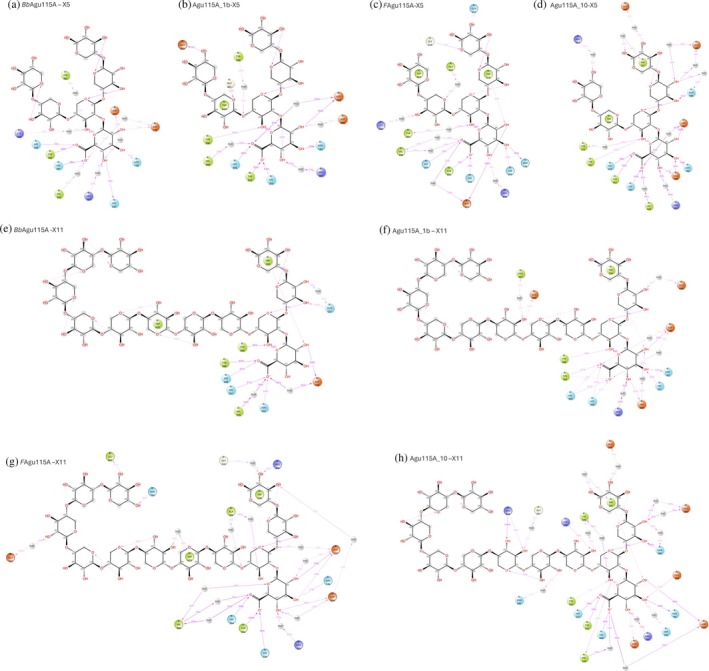
Ligand atom interaction diagrams. Showing interactions that occur more than 30.0% of the 100 ns simulation time in each trajectory. (a) *Bb*Agu115‐X5, (b) Agu115A_1b‐X5, (c) *F*Agu115A‐X5, (d) Agu115A_10‐X5, (e) *Bb*Agu115A‐X11, (f) Agu115A_1b‐X11, (g) *F*Agu115A‐X11, and (h) Agu115A_10‐X11

The polysaccharide preference of *Bb*Agu115A (compared to Agu115A_1b) could be attributed to a mobile loop under the ligand that includes residue Trp620. In all MD simulations of *Bb*Agu115A with X_5_, this Trp‐including loop exhibited high mobility and lost stacking interactions to its corresponding xylose. The same loop was stabilized in presence of X_11_ and facilitated the binding of a longer chain. The stability improvement is also in agreement with the increased *T*_*m*_ of this enzyme in presence of the polysaccharide. This is further supported by the ligand–interaction diagrams, which show strong GlcA recognition for all proteins with both X_5_ and X_11_ ligands (Figure 8). However, the pattern of backbone recognition differed among the enzymes. *Bb*Agu115A shows essentially no aromatic recognition with X_5_ (Figure 8a), whereas *F*Agu115A and Agu115A_10 interact with Trp218 and Trp623 (Figure 8c) and Trp231 and Trp685 (Figure 8d), respectively. Agu115A_1b displays a single aromatic stacking interaction with Trp624 (Figure 8b). Xylose‐chain recognition also appeared to be length‐dependent. For X_11_, *Bb*Agu115A is recognized by both Trp220 and Trp620, while Agu115A_10 shows weaker recognition, stacking only with Trp231. In contrast, *F*Agu115A maintains all its aromatic interactions and is the only enzyme that retains any recognition beyond X_7_, and all these interactions originate from the other protomer (Figure 8g). Similar aromatic recognitions that may guide the polysaccharide backbone into the active site have previously been reported for both four‐ and five‐domain dimers, including cases where the interaction originates from the adjacent protomer (Wang et al., 2016; Wilkens et al., 2022).

**FIGURE 9:**
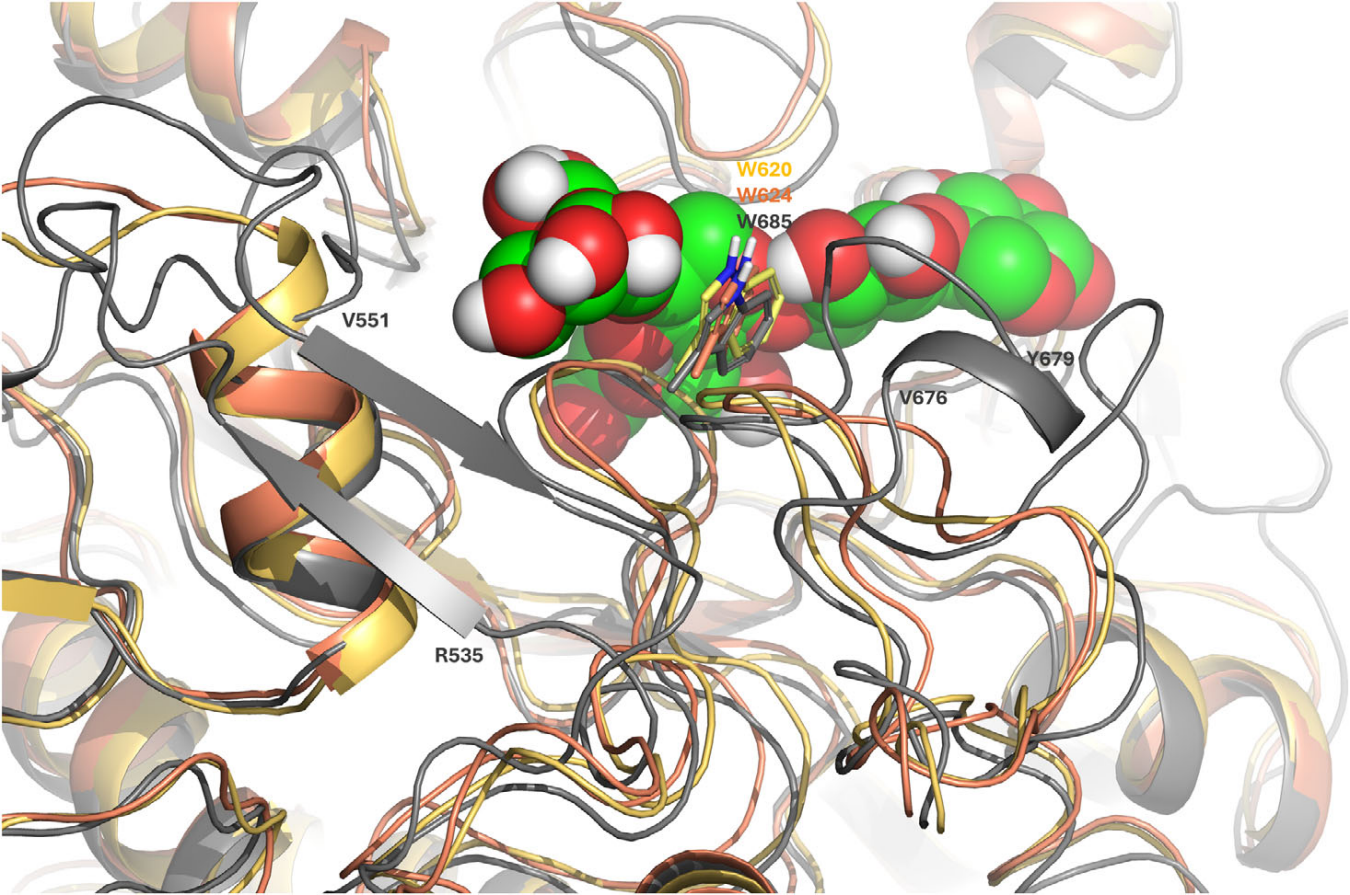
Superimposed structures of BbAgu115A (yellow), Agu115A_1b (orange), and Agu115A_10 (gray) with a X_5_ ligand with GlcA linked to the third xylose moiety showing the β‐strand and half helix present only in Agu115A_10.

Agu115A_10 exhibited the lowest specific activity on the polymer, approximately three times lower than *Bb*Agu115A and 50 times lower than *F*Agu115A. In contrast, it showed a 5.6‐fold higher specific activity on oligomers compared with *Bb*Agu115A, indicating a clear preference of this enzyme for oligomeric substrates. This difference is likely due to two additional insertions in the monomeric Agu115A_10. Like *Bb*Agu115A and Agu115A_1b, it is composed of five domains, but it has a slightly higher molecular weight, increased by approximately 10 kDa. One of these insertions forms a bundle of antiparallel β‐strands, Arg535‐Val551 (Figure 9) located on the lower side of the binding site, which partially hinders accommodation of chains with higher DP than 5. Additionally, a half‐helix forms in the Trp‐loop (Val676‐Tyr679), reducing the overall loop mobility, which is stabilizing the aromatic interactions between Trp685 and the corresponding xylose (Figures 8d and 9).

Analytical gel filtration and OMNISEC showed molecular weights of 113, 220, and 109 kDa for *Bb*Agu115A, *F*Agu115A, and Agu115A_1b, respectively, whereas no defined results were obtained for Agu115A_10. This suggests that *Bb*Agu115A and Agu115A_1b exist as monomers in solution, whereas *F*Agu115A mainly forms dimers. The potential for dimerization was additionally investigated using the AlphaFold server, and the sequences were submitted to be modeled both as monomers and as dimers. All models were successfully generated by AlphaFold (Fig. S7); however, the predicted dimer structures for the two five‐domain *Bb*Agu115A and Agu115A_1b (by wet‐lab data defined as monomers) differed from that of the previously characterized dimeric five domain *Sde*Agu115A (Wang et al., 2016). Specifically, *Bb*Agu115A was predicted to dimerize through an alternative interface, while Agu115A_1b utilized the corresponding loop region for dimerization but with a shifted alignment. Interestingly, FAgu115A, which is a four‐domain enzyme (by wet‐lab data defined as dimeric), also dimerizes in a different way than *Sde*Agu115A, which will be further discussed below. The predicted template modeling (pTM) score and the interface predicted template modeling (ipTM) score were used to estimate model accuracy (Table S1). A pTM score above 0.5 indicates confidence in the predicted model relative to the actual structure, while the ipTM score estimates the accuracy of subunit positioning within a complex, with values above 0.8 indicating high confidence in the prediction (Xu & Zhang, 2010; Zhang & Skolnick, 2004). For *Bb*Agu115A, Agu115A_1b, and *F*Agu115A, the pTM scores of the monomers were 0.95, 0.94, and 0.90, respectively. The corresponding ipTM scores for the dimers were 0.38, 0.53, and 0.91. The same analysis was applied to Agu115A_10, where the wet lab data was inconclusive. The monomer pTM was 0.94, and the dimer ipTM was 0.19, indicating that Agu115A_10 probably exists as a monomer in solution. For the other candidates, these results are consistent with our experimental analyses and further indicate low confidence for dimer formation in *Bb*Agu115A and Agu115A_1b.

Taking all characterized GH115 into consideration, their main differences were observed in the loop regions related to binding, in the presence and location of domain D and the presence of the C^+^ domain (Figure 1). The D domain is superimposed at a matching location in all four‐domain enzymes except for *Bt*GH115A (PDB ID: 5BY3) (Aalbers et al., 2015). *Bt*GH115A differs from the other previously characterized four‐domain GH115 both in substrate specificity (acting on arabinogalactan) and oligomeric state (being a monomer in solution). Taking the structural models into consideration, it becomes apparent that the monomeric proteins (which act as α‐1,2‐ glucuronidases) consist of five distinct domains each, while the dimeric proteins can contain four or five domains. In the four domain *F*Agu115A, each protomer interacts closely with the other through extensive surface contacts. This “shape fit” dimerization creates an interface between the C‐terminal domain of one protomer and the binding site of the other. These close interactions highlight the tendency of four‐domain α‐glucuronidases to dimerize (Figure 6c).

Interestingly, the structure of the five‐domain dimer, *Sde*Agu115A (Wang et al., 2016), resembles that of the monomeric proteins, *Bb*Agu115A, Agu115A_1b, and Agu115A_10, in terms of individual protomer architecture. However, the dimerization mode of *Sde*Agu115A (Wang et al., 2016) is very different from the four‐domain *F*Agu115A, with minimal surface interaction between the protomers. In this case, the two subunits are connected primarily via a long loop, with a three amino acid antiparallel β‐strand. This dimerization pattern is attributed to the C^+^ domain, which may explain the weaker, more transient dimer formation, contrasting with the tight “butterfly” dimer observed in all characterized four‐domain glucuronidases, including the one described in this work (Rogowski et al., 2014; Wilkens et al., 2022).

The dimerization patterns of *SdeAgu115A* (PDB ID: 4ZMH) (Wang et al., 2016) and *AxyAgu115A* (PDB ID: 6NPS) (Yan et al., 2021) are identical. In both crystal structures, the dimer interface is mediated by a three‐amino‐acid β‐sheet, in which a serine occupies the second position. The serine residue from each monomer forms a hydrogen bond with both a conserved asparagine located in the adjacent helix and the corresponding serine from the opposing protomer. This asparagine residue is conserved across all *SdeAgu115A* and *AxyAgu115A* proteins, as well as in the three monomeric proteins.

**FIGURE 10:**
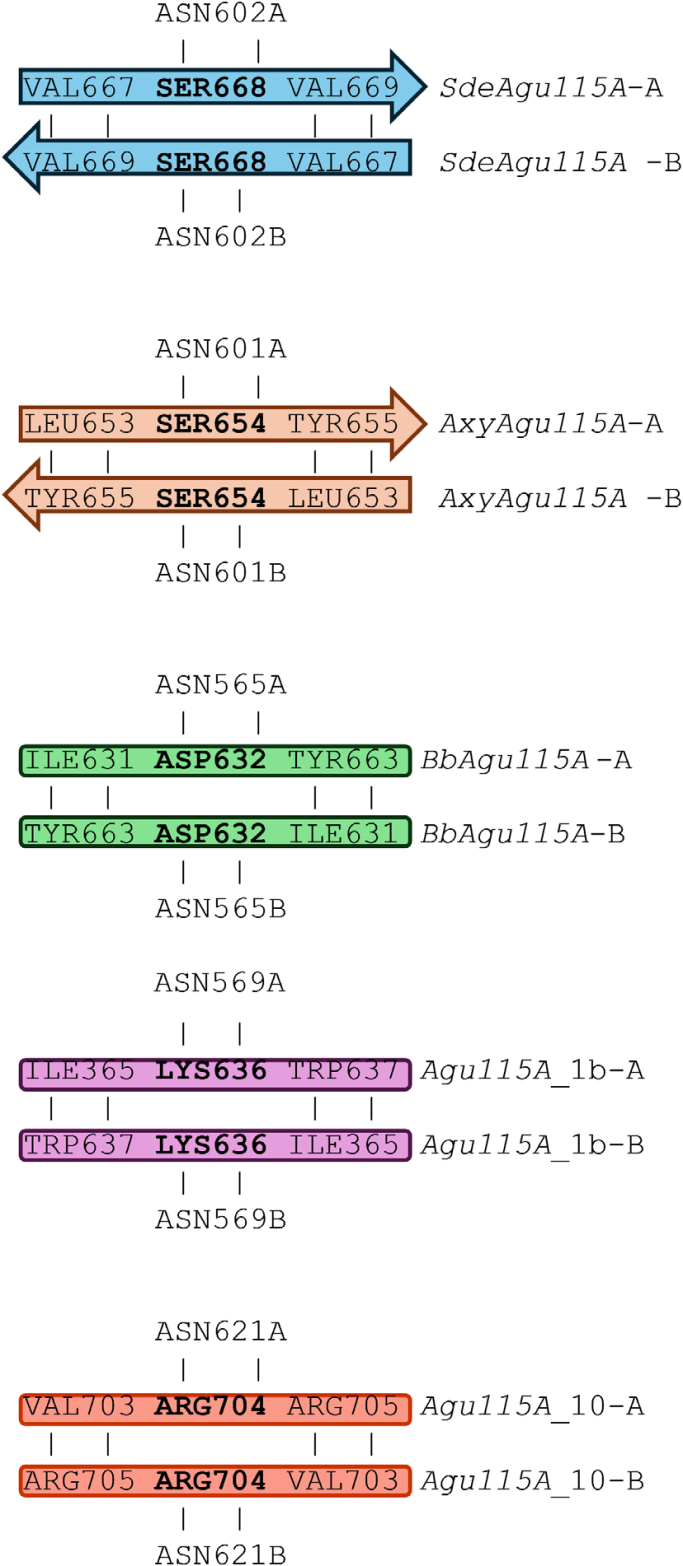
Conserved serine‐mediated hydrogen bonds at the interface of the crystal structures of SdeAgu115A and AxyAgu115A, contrasted with residues unfavorable for dimerization in BbAgu115A, Agu115A_1b, and Agu115A_10.

In *BbAgu115A*, the serine residue is replaced by Asp632. The carboxylate groups from each monomer introduce strong electrostatic repulsion, making dimer formation unlikely. Similarly unfavorable interactions are observed in *Agu115A_1b*, where Ser668 is replaced by Lys636, and in *Agu115A_10*, where the serine is substituted by Arg704 (Figure 10). These substitutions introduce multiple positively charged residues at the interface, further destabilizing the dimer and rendering dimerization unlikely.

## 5 CONCLUSION

In this study, 22 enzymes from GH115 were selected from sequence databases, cloned, and produced in *Escherichia coli*. Although most candidates were detectable in active form in cell extracts, only four were stably soluble following purification. These comprised, one four‐domain enzyme (*F*Agu115A) and three five‐domain enzymes (*Bb*Agu115A, Agu115_1b, and Agu115_10), all functioning as xylan α‐1,2‐glucuronosidases. These four previously uncharacterized CAZymes were examined with respect to sequence features, structural organization, and catalytic function. This work provides the first conclusive evidence for active monomeric xylan α‐1,2‐glucuronosidases within GH115, expanding the current understanding of structural configurations associated with CAZyme functionality. Comparative analyses of monomeric and dimeric proteins revealed clear differences in substrate specificity and catalytic efficiency. To date, all characterized four‐domain GH115 enzymes acting on xylan have been described as dimers. Consistent with this, the four‐domain enzyme characterized in greater detail (*F*Agu115A) was dimeric and displayed preference and superior activity toward polymeric substrates while also showing activity toward oligomeric substrates. By contrast, the five‐domain enzymes were monomeric; one enzyme clearly preferred shorter oligomeric substrates (Agu115_10), one showed little dependence on substrate length, and the third favored longer substrates. Agu115_10 also contained a distinct antiparallel β‐strands bundle insertion that influenced substrate binding, illustrating that domain composition and local structural insertions can alter substrate preference despite conserved catalytic residues. The observed variation in activity among monomeric enzymes indicates that oligomerization is not strictly required for high catalytic efficiency and that domain number does not predict substrate range in a simple linear manner. Overall, these findings refine existing models of structure–function relationships within GH115 and highlight both the structural and evolutionary diversity present in this family.

## AUTHOR CONTRIBUTIONS

**Giang‐Son Nguyen:** Methodology; visualization; writing – review and editing. **Anna Sofia Lewin:** Writing – review and editing; project administration; resources. **Anna Nordborg:** Writing – review and editing; methodology. **Savvina Leontakianakou:** Writing – original draft; conceptualization; investigation; methodology; validation; visualization; formal analysis; data curation. **Lalitha D. Gottumukkala:** Writing – review and editing; funding acquisition; resources. **Anders Sundin:** Visualization; writing – review and editing; supervision; methodology; validation. **Andrius Jasilionis:** Writing – review and editing; methodology. **Carl Grey:** Writing – review and editing; supervision. **Eva Nordberg Karlsson:** Funding acquisition; conceptualization; project administration; writing – review and editing; supervision; resources; investigation. **Simone Balzer Le:** investigation; writing – review and editing; methodology; validation.

## Supporting information


**FIGURE S1.** BbAgu115A model colored on pfam domains. PF15797 domain, residues 164‐496 is showing in orange and PF17829, residues 752‐920 in yellow.
**FIGURE S2.** Domain chart of all the candidates evaluated in this work and all known GH115s.
**FIGURE S3.** Overlayed alpha‐fold models of OtAgu115_2 and PsAgu115A to the crystal structure of 5BY3, showing similar domain folding.
**FIGURE S4.** Overlayed alpha‐fold models of PtAgu115A (light green), WfAgu115A (purple), Agu115A_3 (blue), Agu115A_6 (dark blue). Agu115A_8 (dark green) to FAgu115A (lime) the only one with D‐domain, indicated by a circle.
**FIGURE S5.** Overlayed alpha‐fold models of BbAgu115A (purple) and Agu115A_4 (gray) showing the additional domains present in the later.
**FIGURE S6.** Overlayed alpha‐fold models of FAgu115A (lime) and Agu115A_2 (cyan) with D domain and E domain located at opposite end respectively.
**TABLE S1.** AlphaFold3 pTM and ipTM scores for BbAgu115A, Agu115A_1b, FAgu115A, and Agu115A_10 for monomer and dimer prediction.
**FIGURE S7.** AlphaFold3 models of the four proteins characterized in this study. The color indicates the degree of confidence of the model. The same loop region exhibits some uncertainty in all candidates.

## Data Availability

The data that support the findings of this study are available in CAZy at https://www.cazy.org/. These data were derived from the following resources available in the public domain: GenBank, https://www.ncbi.nlm.nih.gov/genbank/.
