# Supplementary material for: Novel GH115 xylan α‐1,2‐glucuronidases with distinct preferences for xylan‐derived oligomers and polymers
Source: Protein Sci. 2026 Apr 8;35(5):e70571. doi: 10.1002/pro.70571 (PMC13059095; doi:10.1002/pro.70571)
Supplement: Supplementary file 1 — FIGURE S1. BbAgu115A model colored on pfam domains. PF15797 domain, residues 164‐496 is showing in orange and PF17829, residues 752‐920 in yellow. FIGURE S2. Domain chart of all the candidates evaluated in this work and all known GH115s. FIGURE S3. Overlayed alpha‐fold models of OtAgu115_2 and PsAgu115A to the crystal structure of 5BY3, showing similar domain folding. FIGURE S4. Overlayed alpha‐fold models of PtAgu115A (light green), WfAgu115A (purple), Agu115A_3 (blue), Agu115A_6 (dark blue). Agu115A_8 (dark green) to FAgu115A (lime) the only one with D‐domain, indicated by a circle. FIGURE S5. Overlayed alpha‐fold models of BbAgu115A (purple) and Agu115A_4 (gray) showing the additional domains present in the later. FIGURE S6. Overlayed alpha‐fold models of FAgu115A (lime) and Agu115A_2 (cyan) with D domain and E domain located at opposite end respectively. TABLE S1. AlphaFold3 pTM and ipTM scores for BbAgu115A, Agu115A_1b, FAgu115A, and Agu115A_10 for monomer and dimer prediction. FIGURE S7. AlphaFold3 models of the four proteins characterized in this study. The color indicates the degree of confidence of the model. The same loop region exhibits some uncertainty in all candidates. [file PRO-35-e70571-s001.docx]

Novel GH115 xylan α-1,2-Glucuronidases with distinct preferences for xylan-derived oligomers and polymers

Savvina Leontakianakou*^a^, Simone Balzer Le^b^, Anders Sundin^c^, Andrius Jasilionis^a^, Giang-Son Nguyen^b^, Anna Nordborg^b^, Lalitha D. Gottumukkala^d^, Carl Grey^a^, Anna Sofia Lewin^b^, Eva Nordberg Karlsson^a^


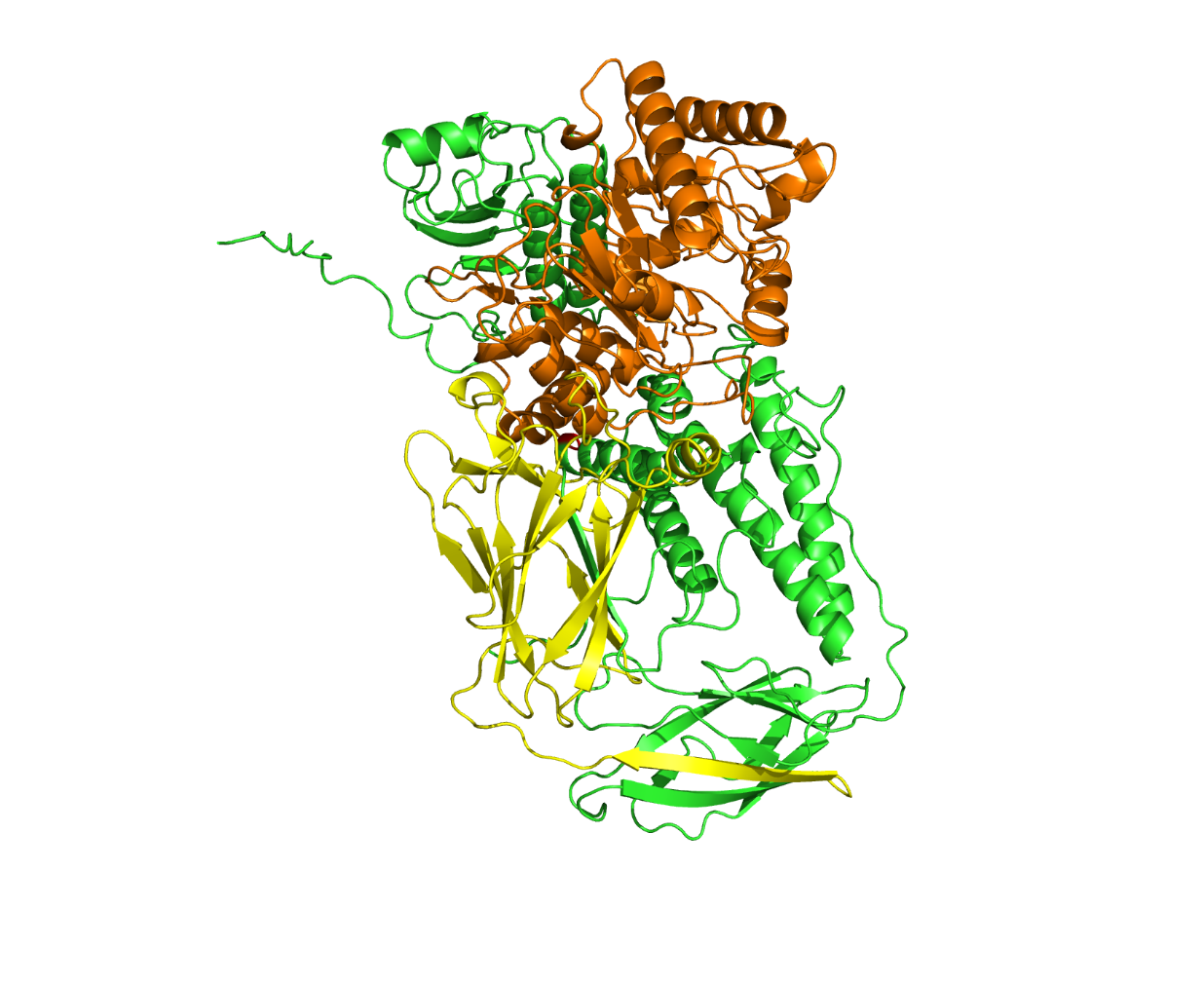


Figure 1. BbAgu115A model coloured on pfam domains. PF15797 domain, residues 164-496 is showing in orange and PF17829, residues 752-920 in yellow.

Figure 2. Domain chart of all the candidates evaluated in this work and all known GH115s


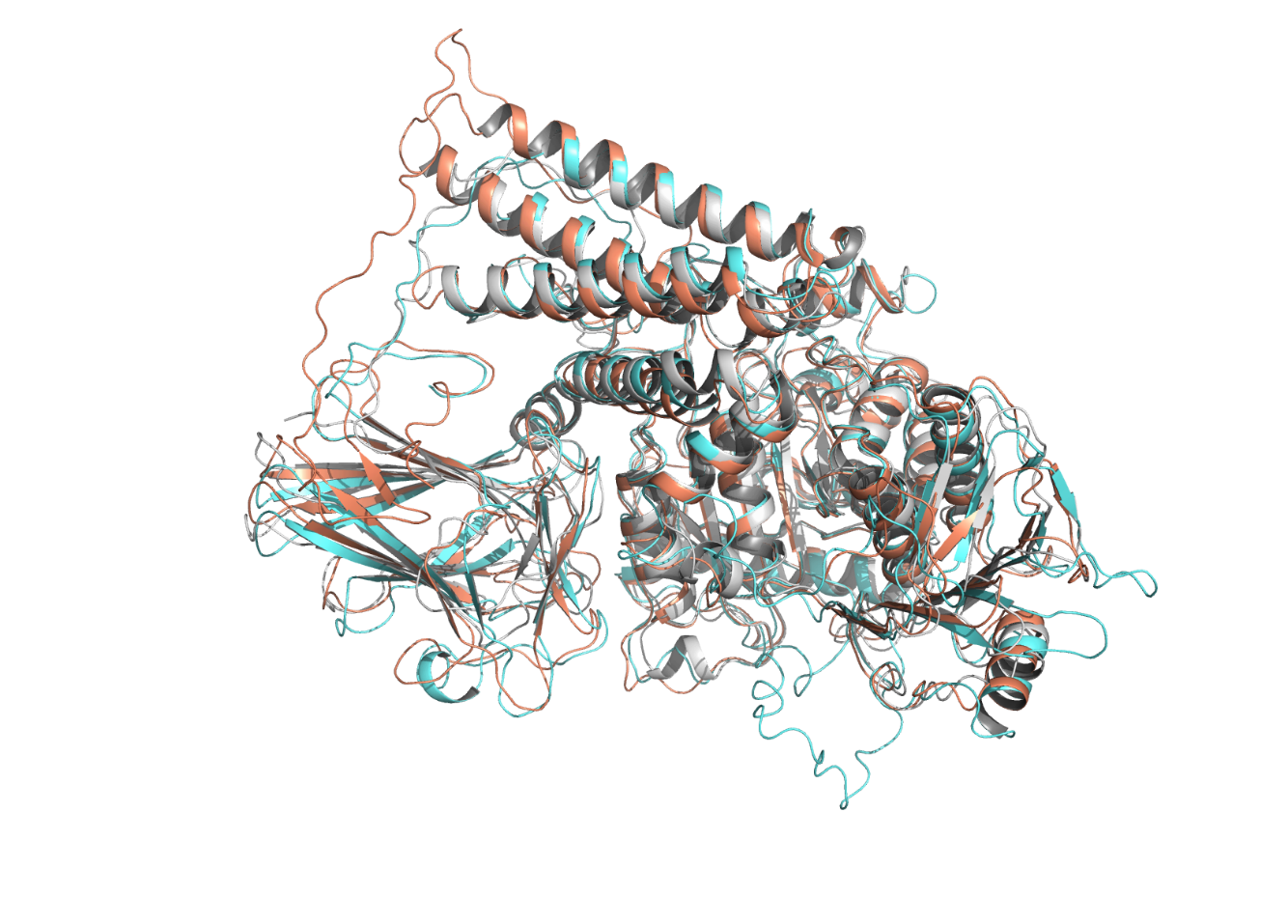


Figure 3. Overlayed alpha-fold models of OtAgu115_2 and PsAgu115A to the crystal structure of 5BY3, showing similar domain folding.

Figure 4. Overlayed alpha-fold models of PtAgu115A (light green), WfAgu115A (purple), Agu115A_3 (blue), Agu115A_6 (dark blue). Agu115A_8 (dark green) to FAgu115A (lime) the only one with D-domain, indicated by a circle.


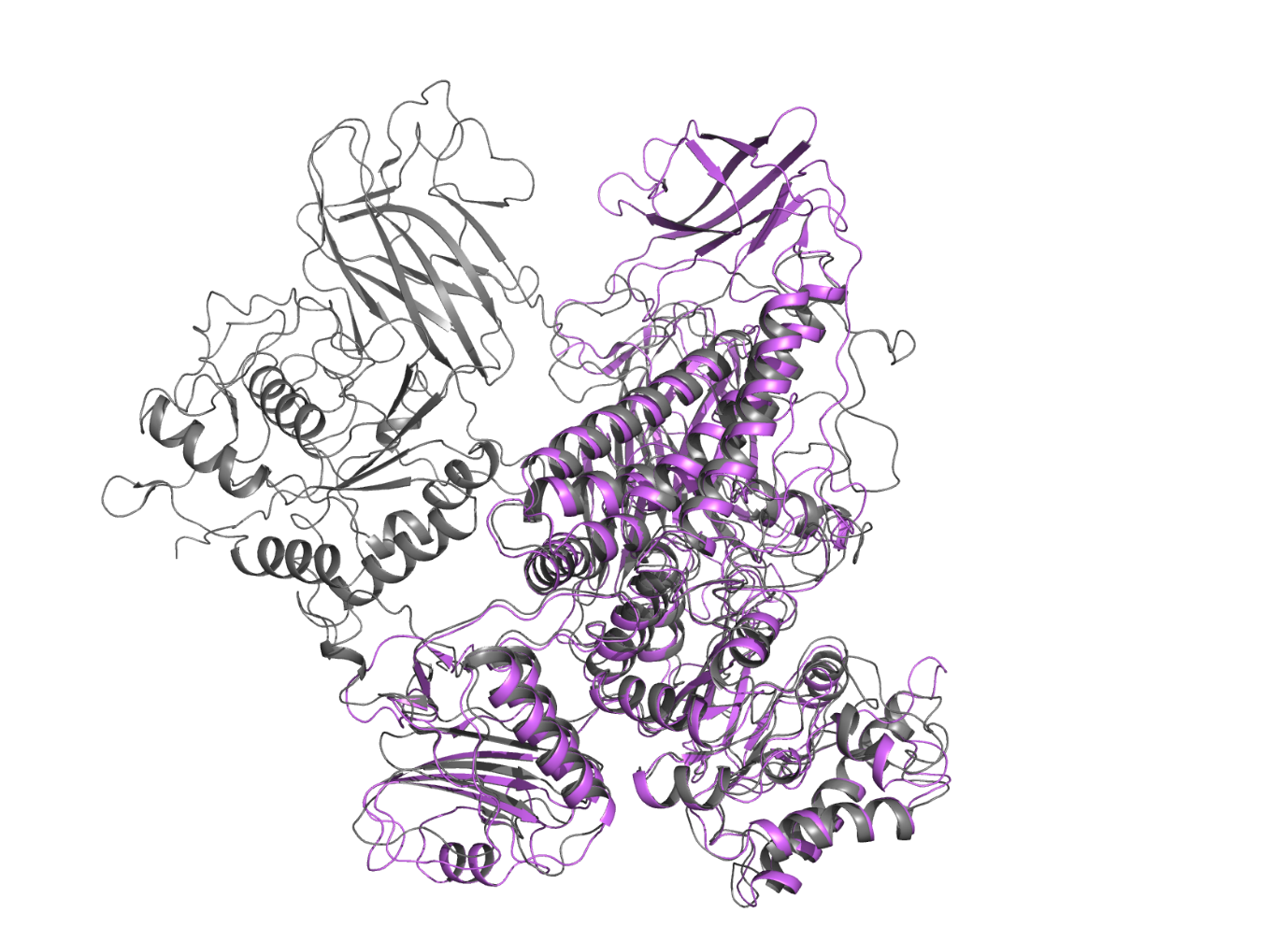


Figure 5. Overlayed alpha-fold models of BbAgu115A (purple) and Agu115A_4 (grey) showing the additional domains present in the later.


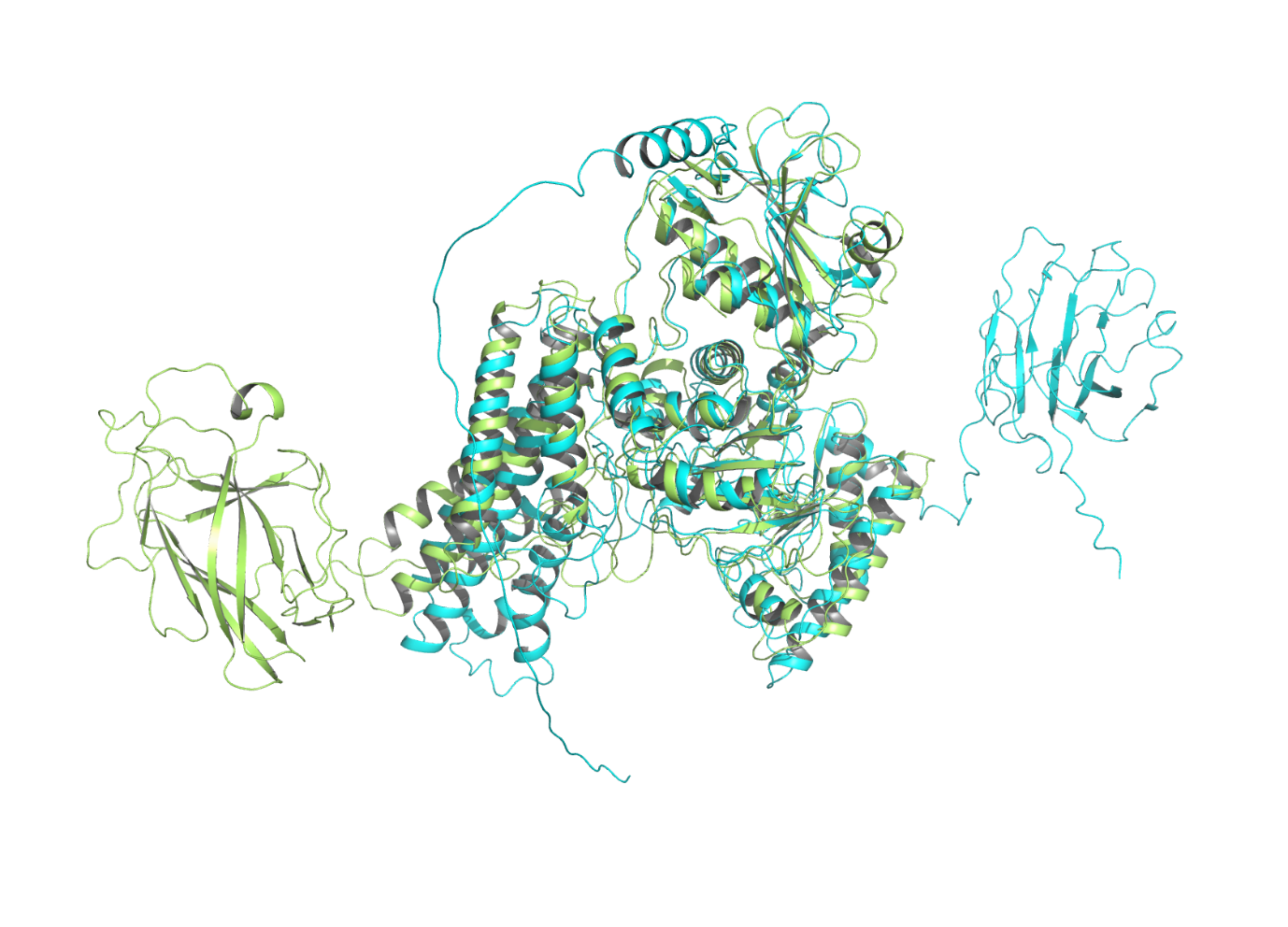


Figure 6. Overlayed alpha-fold models of FAgu115A (lime) and Agu115A_2 (cyan) with D domain and E domain located at opposite end respectively.

Table 1. AlphaFold3 pTM and ipTM scores for BbAgu115A, Agu115A_1b, FAgu115A, and Agu115A_10 for monomer and dimer prediction.

| Enzyme | Monomer | Dimer | |
| --- | --- | --- | --- |
|  | pTM | pTM | ipTM |
| *Bb*Agu115A | 0.95 | 0.67 | 0.38 |
| Agu115A_1b | 0.94 | 0.74 | 0.53 |
| *F*Agu115A | 0.9 | 0.9 | 0.91 |
| Agu115A_10 | 0.94 | 0.53 | 0.19 |

Figure 7. AlphaFold3 models of the four proteins characterised in this work. The colour indicates the degree of confidence of the model. The same loop region exhibits some uncertainty in all candidates.
